# DNA glycosylases Ogg1 and Mutyh influence gene expression of PRC2 targets associated with cognition

**DOI:** 10.1007/s00018-025-05730-9

**Published:** 2025-08-08

**Authors:** Andreas Abentung, Teri Sakshaug, Rabina Dumaru, Nina-Beate Liabakk, Mingyi Yang, Junbai Wang, Magnar Bjørås, Katja Scheffler

**Affiliations:** 1 Department of Clinical and Molecular Medicine, Faculty of Medicine and Health Sciences, Norwegian University of Science and Technology (NTNU), Trondheim, Norway; 2 Department of Neuromedicine and Movement Science, Faculty of Medicine and Health Sciences, Norwegian University of Science and Technology (NTNU), Trondheim, Norway; 3 Department of Neurology and Clinical Neurophysiology, University Hospital Trondheim, Trondheim, Norway; 4 Department of Microbiology, Oslo University Hospital HF, Rikshospitalet and University of Oslo, Oslo, Norway; 5 Department of Clinical Molecular Biology (EpiGen), University of Oslo and Akershus University Hospital, Akershus, Norway; 6 Centre for Embryology and Healthy Development, University of Oslo, Oslo, Norway

**Keywords:** DNA glycosylase, PRC2, Epigenome, Transcription, Cognition

## Abstract

Base excision repair, initiated by DNA glycosylases, preserves genomic integrity by removing damaged bases. DNA glycosylases Ogg1 and Mutyh were shown to alter the hippocampal transcriptome independently of DNA damage repair. However, the role of DNA glycosylases as transcriptional regulators and their involvement in cognition remain unclear. Here, we combine transcriptomic and epigenomic analyses of the hippocampus in mice deficient in DNA glycosylases. We report that the combined deficiency of Ogg1 and Mutyh impairs spatial long-term memory. Mechanistically, Ogg1 and Mutyh modulate DNA methylation of polycomb repressive complex 2 (PRC2) target genes. PRC2 occupancy and associated histone post-translational modifications depend on Ogg1 and Mutyh in neurons and glia. These epigenetic changes correlate with cell-type specific differences in gene expression of PRC2 targets. Finally, human genetic data link DNA glycosylase-regulated genes to cognitive function, highlighting a novel role for Ogg1 and Mutyh in modulating the epigenome to control transcriptional responses relevant for brain-related diseases.

**Graphical Abstract:**

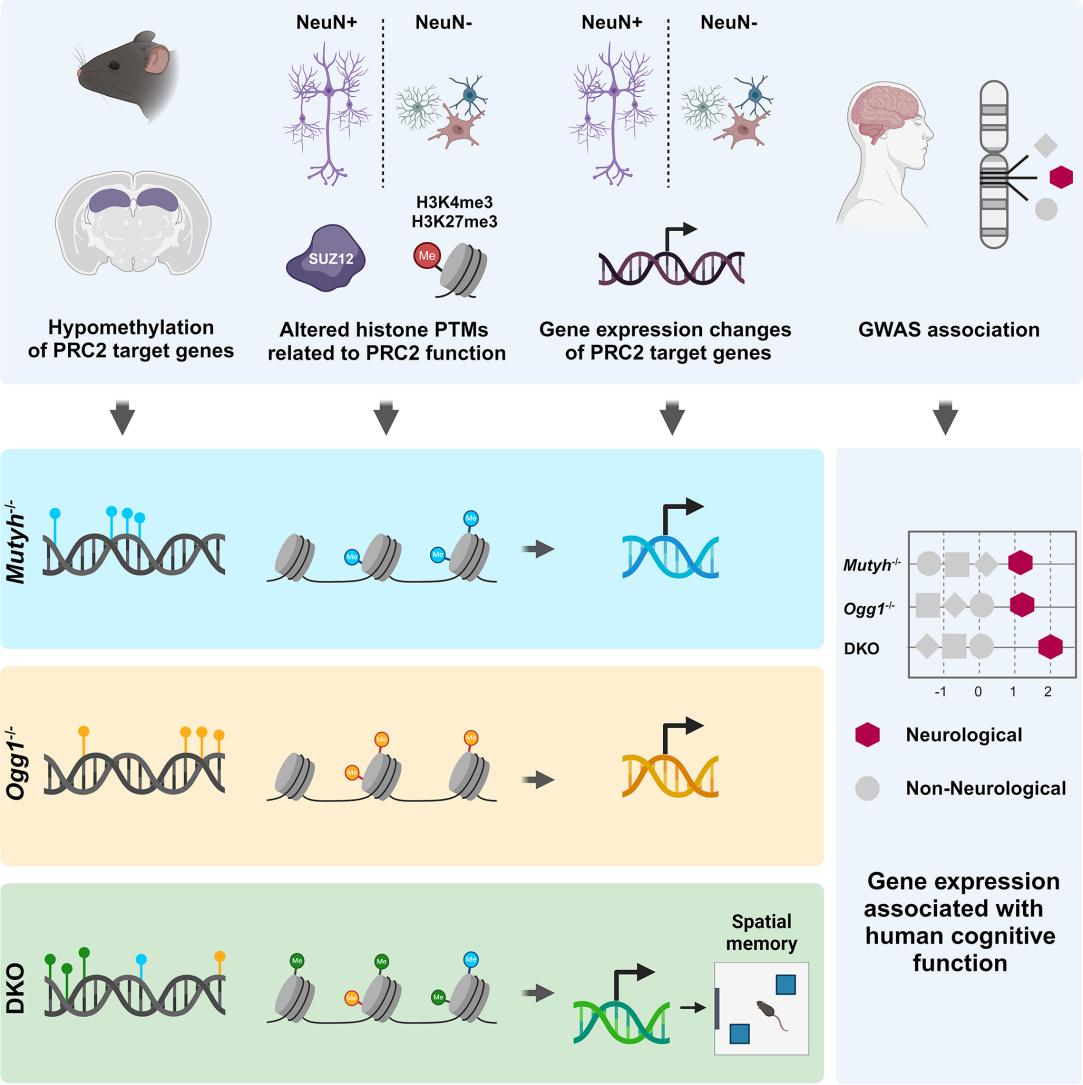

Created in BioRender. Scheffler, K. (2025). https://BioRender.com/jwscej4.

**Supplementary Information:**

The online version contains supplementary material available at 10.1007/s00018-025-05730-9.

## Introduction

The canonical functions of DNA repair play an essential role in maintaining genomic integrity and stability across organisms [1]. Genomic instability caused by an accumulation of DNA damage is a hallmark of aging [2] and has been linked to DNA mutations, altered gene expression and cognitive impairment in the human brain [3, 4]. Due to its high metabolic rate, high transcriptional activity and long lifespan, brain cells are highly susceptible to DNA damage [5]. Consistently, mutations in DNA repair genes and impaired DNA repair pathways are associated with pre-mature aging and various neurodegenerative diseases [6, 7]. Base excision repair (BER) initiated by DNA glycosylases is the major pathway for removal of oxidized bases in the DNA [8]. Given its low redox potential, the DNA base guanine is particularly susceptible to oxidation, leading to the formation of the pre-mutagenic base lesion 8-oxoguanine (8-oxoG) [9]. 8-oxoG DNA glycosylase 1 (Ogg1) recognizes and facilitates the removal of 8-oxoG within the genome through BER [8, 10]. Adenine DNA glycosylase (Mutyh) excises mis-incorporated adenine opposite of 8-oxoG during replication, allowing Ogg1 to remove 8-oxoG after insertion of the correct base [11].

Emerging evidence indicates that DNA glycosylases may have additional functions beyond canonical DNA repair by altering the epigenetic landscape and thereby regulating transcription [12]. In the mouse brain, Ogg1 and Mutyh regulate gene expression related to learning and memory independent of global accumulation of 8-oxoG [13]. In line with these findings, catalytically inactive Ogg1 promotes pro-inflammatory gene expression in other cell-types, highlighting the significant role of DNA glycosylases beyond 8-oxoG repair [14]. Moreover, several DNA glycosylases were identified as potential readers of epigenetic DNA modifications [15, 16] and have been implicated in active DNA demethylation [17, 18]. Additionally, DNA glycosylases have been shown to interact with several epigenetic modifiers such as DNA methyltransferases [19, 20], histone demethylases [21] and members of the polycomb repressor complex 2 (PRC2) [22].

It has also been demonstrated that 8-oxoG itself exhibits epigenetic-like properties by modulating gene transcription through its accumulation in promoter regions [23, 24]. Recent evidence indicates that both 8-oxoG and DNA glycosylases alter gene expression independently of one another [25]. Nevertheless, the molecular mechanisms through which DNA glycosylases affect brain health, particularly in relation to cognition, are still not fully understood.

In this study, we investigate the impact of Ogg1 and Mutyh on the epigenetic landscape in the adult mouse hippocampus. We show that loss of DNA glycosylases affects spatial but not associative long-term memory. Mechanistically, we demonstrate that Ogg1 and Mutyh influence gene expression by modulating DNA methylation levels at regulatory regions of predominantly PRC2 target genes, as well as histone modifications linked to PRC2 activity, in both neurons and glia. In human genetic data, we find genes regulated by DNA glycosylases to be enriched for common variants associated with cognitive function and brain-related diseases. Taken together, our results reveal a previously unrecognized role of DNA glycosylase beyond DNA repair by mediating gene expression important for higher human brain function and health.

## Materials and methods

### Animals

Mice were housed and bred at the Comparative Medicine Core Facility (Trondheim, Norway) in a 12-hour light/dark cycle with access to food and water *ad libitum* under standard laboratory conditions of 19–22 °C with air humidity of 50–60%. Ogg1 and Mutyh deficient C57BL/6N mice were used for all the experiments included in this study. Double knockout (DKO) mice were obtained by crossing *Ogg1*^*-\-*^ and *Mutyh*^*-\-*^ mice and wild type (WT) C57BL/6N mice were used as control groups. For all experiments 6 months old male mice were used. Mouse litters were genotyped using genomic DNA obtained from ear biopsies. All knockout strains have been previously described elsewhere [13].

### Contextual fear conditioning

Mice were handled 5 min for three days prior to the experiment. Contextual fear conditioning was performed in a 17 × 17 × 25 cm chamber with transparent walls and a metal rod floor (Ugo Basile, Fear Conditioning Animal System, 46001), cleaned with soap-water and illuminated to 150 lx, as previously described [26]. After a 120 s acclimation period, mice were conditioned with three presentations of a 0.60 mA scrambled foot shock, with a 120 s inter-shock interval. The mice were allowed to remain in the chamber for an additional 120 s following the last stimulus presentation. Short-term and long-term fear memories were assessed by re-exposing the mice for 300 s either 1 h or 24 h later to the conditioning chamber without the administration of a foot shock. Freezing was measured as an index of fear [27], defined as no visible movement except that required for respiration. Freezing time was scored and analyzed with Any-Maze software (Stoelting Europe) and converted to a percentage [(duration of freezing /total time) × 100].

### Flinch-jump test

Reactivity to the foot shock was evaluated in the same apparatus used for contextual fear conditioning. After a 120 s acclimation period, mice were subjected to a series of foot shocks with gradually increasing amperage (0.1 mA every 30 s) starting from 0.1 mA. Mice were scored for their first visible response to the shock (flinch), their first pronounced motor response (run or jump) and their first vocalized distress, as previously described [28].

### Novel object location

Animals were housed under a 12-hour reversed light/dark cycle for at least a week before the start of the paradigm. Prior to training, mice were handled 5 min for five consecutive days and then habituated to the experimental apparatus (50 × 50 × 30 cm open field area) for 5 min for three consecutive days in the absence of objects, as previously described [26]. During training, mice were placed into the experimental apparatus containing two identical objects (Duplo blocks) and allowed to explore for 10 min. Short-term and long-term retention tests were conducted by placing the mice back into the experimental apparatus for 10 min either 1 h or 24 h after training. To assess spatial object location-dependent memory, the placement of one familiar object remained unchanged whereas the other familiar object was moved and placed adjacent to a different corner inside the experimental apparatus. Exploration was scored when the head of the animal entered the virtual zone adjacent to the object. All training and testing trials were videotaped and analyzed with Any-Maze software (Stoelting Europe). The relative exploration time (t) was expressed as a percent discrimination index (D.I. = tnovel / (tnovel + tfamiliar) × 100%). Mean exploration times were then calculated and the discrimination indices between experimental groups were compared. Animals that explored each object for less than 3 s during training or explored both objects for less than 10 s total during testing were excluded from the analysis.

### Tissue collection

Animals were sacrificed by cervical dislocation and hippocampi were dissected, isolated, snap-frozen in liquid nitrogen and stored at − 80 °C until further use. All mice included in high throughput sequencing experiments were re-genotyped by using genomic DNA obtained from tail biopsies after being sacrificed.

### DNA isolation

Total DNA was extracted from snap-frozen hippocampi by using AllPrep DNA/RNA/Protein mini kit (Qiagen, 80004), according to manufacturer’s protocol. Frozen tissue was transferred to tubes with 1.4 mm stainless steel beads containing 350 ml buffer RLT and homogenized using a Roche MagNA Lyser rotor (5000 rpm, 10 s). Samples were further processed according to the respective protocol. DNA concentrations were measured by using a ND-1000 spectrophotometer (Nanodrop Technologies).

### Whole genome bisulfite-sequencing

Total DNA from two replicates of each genotype was sent to BGI Tech Solutions Co., Hong Kong. Bisulfite conversion, library preparation and DNA sequencing was performed by BGI as previously described [29]. Briefly, total hippocampal DNA was fragmented by sonication using a Bioruptor (Diagenode) to a mean size of approximately 250 bp, followed by blunt-ending and dA addition to 3’-end. Finally, adaptor ligation (in this case, with methylated adaptors to protect from bisulfite conversion) was performed according to the manufacturer’s instructions. Ligated DNA was bisulfite converted using the EZ DNA Methylation-Gold kit (Zymo research, D5005). After treatment with sodium bisulfite, unmethylated cytosine residues were converted to uracil whereas 5-methylcytosine (5mC) remains unaffected. Different insert size fragments were excised from the same lane of a 2% TAE agarose gel. Products were purified by using QIAquick Gel Extraction kit (Qiagen, 28704) and amplified by PCR. After PCR amplification, uracil residues were converted to thymine. Pooled libraries were sequenced (paired-end, 100 bp in length) on a BGISEG-500 sequencer with an average depth of 1.3 billion reads per sample.

### Fluorescence activated nuclear sorting (FANS)

Hippocampal nuclei were extracted, isolated and sorted as previously described, with some minor modifications [30]. In brief, snap-frozen hippocampi from 5 animals of the same genotype were pooled in 1 ml of low sucrose buffer [(0.32 M sucrose, 10 mM HEPES pH 8.0, 5 mM CaCl_2_, 3 mM Mg(CH_3_COO)_2_, 0.1 mM EDTA, 0.1% Triton X-100, 1 mM DTT, 1x Complete EDTA free protease inhibitor cocktail (Roche, 11873580001) and 20 U/ml of RNase Inhibitor (Applied Biosystems, N8080119)]. All steps except the crosslinking were performed at 4 °C or on ice. Hippocampal tissue was homogenized by applying pestle “loose” and “tight” 40 times, respectively. For nuclei fixation, formaldehyde was added at a final concentration of 1% and the samples were incubated at room temperature for 10 min on a rotator. The crosslinking reaction was stopped by adding glycine to a final concentration of 125 mM for 5 min at room temperature. Nuclei were then pelleted by centrifugation, re-suspended and washed twice with 1 ml of low sucrose buffer. Subsequently, nuclei were purified through a sucrose cushion (10 mM HEPES pH 8.0, 1 M sucrose, 3 mM Mg(CH_3_COO)_2_, 1 mM DTT, 6 ml of cushion for 1.5 ml of lysate) by centrifugation at 3200 g for 10 min in a 15 ml Falcon tube. Afterwards, nuclei were re-suspended in nuclei buffer (PBS, 0.1% Tween 20 in PBS, 5% BSA, 1x Complete EDTA free protease inhibitor cocktail (Roche, 11873580001) and 20 U/ml of RNase Inhibitor (Applied Biosystems, N8080119)) containing 3% goat serum and cleared by filtering through a 40 μm cell strainer. The nuclei suspension was stored on 4 °C overnight. On the next day, nuclei were stained with anti-NeuN mouse antibody (Millipore, mab377) diluted 1:500 for 60 min at 4 °C. The samples were then washed twice with nuclei buffer containing 3% goat serum. Subsequently, the samples were stained for 60 min at 4 °C with a secondary antibody (Alexa 488, Life Technologies, A11001) diluted 1:1000. Nuclei were then washed twice with nuclei buffer and stored until sorting. Directly before sorting, nuclei were dissociated by passing them 10 times through a 25G needle and by filtering through a 40 μm cell strainer into ice-cold conical tubes containing 1.8 ml of filtered PBS-BSA (5%). Lastly, 2 µl of Vybrant™ DyeCycle™ Ruby (V10273, Life Technologies) was added to the nuclei suspension. Nuclei were sorted using an 85 μm nozzle on a FACSAria II (BD Bioscience). Nuclei selection was based on DyeCycle Ruby staining, singlet nuclei detection and NeuN + positive staining. Both NeuN stained (NeuN+) and unstained (NeuN−) fractions were collected. The average purity of the sorted nuclei exceeded 95%, yielding highly cell-type specific material. The NeuN positive population contains every neuron expressing NeuN endogenously, which are primary excitatory neurons as well as interneurons. NeuN negative cells consist predominantly of glial cells but also contains other cell-types. Flow cytometry data were analyzed and plotted by using FlowJo (BD Bioscience).

### Chromatin Immunoprecipitation (ChIP)

Sorted nuclei were pelleted by centrifugation at 3200 g for 15 min, transferred to Bioruptor Microtubes (Diagenode, C30010016) shearing tubes and carefully re-suspended in RIPA buffer (10 mM Tris-Cl, pH 8.0, 140 mM NaCl, 1 mM EDTA, 1% Triton X-100, 0.1% sodium deoxycholate, 1% SDS and 1x Complete EDTA free protease inhibitor cocktail (Roche, 11873580001). Samples were incubated for 10 min at 4 °C and then sheared using a Bioruptor (Diagenode) 4 times at 5 cycles and followed by 2 cycles at 30″ ON/OFF high power. Samples were spun down in between every 5 cycles. Sheared chromatin was cleared by centrifugation at 10000 rpm for 10 min and the supernatant was stored in DNA low-binding tubes (Eppendorf, 022431021). To confirm the successful fragmentation of chromatin, DNA from a small aliquot of each sample was purified by phenol-chloroform extraction. The size of the DNA fragments was analyzed through gel electrophoresis on a 1.6% Agarose gel with GelRed^®^ nucleic acid stain (Biotium, 41003) and the DNA concentration was determined by a ND-1000 spectrophotometer (Nanodrop Technologies). The rest of the chromatin was snap-frozen and stored at 80 °C until further use. For chromatin immunoprecipitation, sheared chromatin was pre-cleared with BSA-blocked protein G magnetic beads (Dynabeads, Invitrogen, 10004D) for 1 h at 4 °C. For every ChIP-reaction the appropriate amount of chromatin, depending on the different antibodies, was used (Supplementary Table 1). ChIP-reactions were performed by overnight incubation on a rotating wheel at 4 °C in ChIP buffer (50 mM Tris-HCl at pH 8.0, 150 mM NaCl, 1% NP-40, 0.5% sodium deoxycholate, 20 mM EDTA, 1x Complete EDTA free protease inhibitor cocktail). As input control a 10% aliquot of each sheared chromatin sample was saved and stored at -80 °C. Subsequently, 30 µl of BSA-blocked protein G magnetic beads were added to each ChIP-reaction and the mixture was incubated on a rotator at 4 °C for 4 h. The chromatin-bead complexes were washed twice at 4 °C for 5 min at slow rotation (15 rpm) with ChIP buffer containing 0.1% SDS, twice with wash buffer (100 mM Tris-HCl pH 8.0, 500 mM LiCl, 1% NP-40, 1% sodium deoxycholate, 20 mM EDTA) and twice with TE buffer (10 mM Tris-HCl pH 8.0, 1 mM EDTA). For reverse crosslinking, chromatin-bead complexes and 10% input controls were resuspended in 300 µl elution buffer (100 mM NaHCO_3_, 1% SDS) with 200 mM NaCl and incubated at 65 °C overnight. On the next day, the samples were treated with 1 µl of RNAse A (Thermo Scientific, EN0531) for 1 h at 37 °C. Subsequently, 6 µl 0.5 M EDTA and 1 µl Proteinase K (Roche, 3115828001) were added and the samples were incubated at 45 °C for 1 h. DNA was then purified by phenol-chloroform extraction and samples were eluted in 16 µl Tris buffer (10 mM Tris-HCl pH 8.0) in a DNA low-binding tube and stored at − 80 °C.

### ChIP-sequencing

DNA concentrations for ChIP reactions and inputs were determined by using a Qubit dsDNA HS Assay Kit (Thermo Fisher, Q32851). Library construction was performed by using QIAseq™ Ultralow Input Library Kits (Qiagen, 180492) according to manufacturer’s protocol. The final libraries were purified by using AMPure XP beads (Beckman Coulter, A63880) and eluted in 23 µl nuclease free water. DNA fragment size was determined by using an Agilent 2100 Bioanalyzer and the concentration of the final libraries was measured with a Qubit dsDNA HS Assay Kit (Thermo Fisher, Q32851). Libraries were further validated by qPCR using a KAPA Library Quantification Kit (Roche, KK4824) and pooled before being sequenced. Sequencing (single-end, 75 bp) was performed at the Genomic Core Facility (GCF, Trondheim) using an Illumina NextSeq500 sequencer with an average depth of 20 million reads per sample.

### Nuclear RNA-sequencing

Hippocampal nuclei were pooled from 3 animals per genotype. Nuclear RNA of NeuN + and NeuN- nuclei was isolated by using the RNeasy^®^ FFPE Kit (Qiagen, 73504) according to manufacturer’s protocol. RNA concentration and quality was determined using Qubit RNA Assay Kits (Thermo Fisher, Q32852 and Q10210) and an Agilent 2100 Bioanalyzer, respectively. Libraries were prepared by using a SMARTer^®^ Stranded Total RNA-Seq Kit (Takara, 634485) according to the manufacturer’s instructions. Libraries were pooled and sent to BGI Tech Solutions Co., Hong Kong. Sequencing (paired-end, 100 bp) was performed using a MGISEQ2000 sequencer with an average depth of 50 million reads per sample.

### Genomic data processing

#### Bisulfite-seq data

After trimming and FastQC analysis, the clean GWBS data were aligned to the mouse reference genome GRCm38, followed by a separate extraction of genome-wide 5mC in the context of CG, CHG and CHH by using Bismark [31] (version 0.22.3). The 5mC count data was cleaned by filtering out low reads of coverage < 4 in each cytosine. The differentially methylated regions (DMRs) were identified by HMST-Seq-Analyzer [32]. In brief, methylation regions were extracted from predefined genomic areas (e.g. TSS, TES, and gene body) and DMRs were predicted by robust statistical tests (Wilcoxon rank-sum test) between samples. The parameter setting was set to default for gene feature regions, requiring at least 3 mC sites in the TSS and TES, 5mC sites in the gene body, and a maximum distance of 200 bp between adjacent mC sites within a region. Since HMST-Seq-Analyzer is only suited for two sample comparison: replicates of all knockouts (KO1, KO2) were compared to both replicates from WT control samples. The common DMRs among the 4 pair-wise comparisons were defined as the true DMRs. The methylation difference is presented as relative ratio (rratio), which is defined by: < methylation level in KO>– < methylation level in WT> ) / < mean methylation of KO and WT>. DMRs were filtered by rratio > 0.1 as common hyper, and by rratio < -0.1 as common hypo. The methylation profile plots were generated by ggplot2 (version 3.4.0) using the default smoothing method of auto detection in R package. Analysis of upstream regulators of DMRs was done using Ingenuity Pathway Analysis (IPA; Qiagen) and PRC2 gene lists were exported for profile plots in ggplot2.

#### ChIP-seq data

All datasets were quality assessed using FastQC and FastQ Screen. Fastq reads were aligned to the GRCm38/mm10 genome using Bowtie2 [33] and files were processed using samtools [34]. Visualization of bigwigs for genome tracks and profile plots were generated from bam files using deepTools [35]. Correlation heatmap and profile plots were generated using deepTools and profile plot values were exported and analyzed using Graph Pad Prism (GraphPad Software, Inc., La Jolla, CA, v10). Peak calling was performed using epic2 [36] with an FDR cutoff of 0.05. Window and gap sizes were 100 bp, 3 gaps for H3K4me3, 200 bp and 3 gaps for H3K27me3 and 50 bp, 2 gaps for Suz12. Reads were counted over consensus peaks, which were defined as peaks present in at least two of three replicates, using the Bioconductor package DiffBind (v.3.10.1) in R (v.4.3.2). Differential enrichment analysis was performed using edgeR (v.3.42.4) between genotypes. Differentially enriched regions (DERs) were annotated to the closest gene and assigned to genomic features using ChIPseeker [37] (v1.36). Volcano plots were generated using the Bioconductor package EnhancedVolcano (v. 1.18.0) in R where differentially enriched regions were defined as having a p-value < 0.05 and an absolute log2FC > 0.5. Pathway analysis for DERs was performed using IPA (Qiagen), pathway heatmaps were made using GraphPad Prism (GraphPad Software, Inc., La Jolla, CA, v10) and all Venn diagrams were made using Venny [38]. RPKM values were counted over all DERs using DiffBind and exported to Perseus [39] where values were z-scored and clustered using average linkage and Euclidean distance.

#### RNA-seq data

RNAseq datasets were quality assessed using FastQC and FastQ Screen before processing. Paired-end reads were aligned to the GRCm38/mm10 genome using Hisat2 [40] and files were processed using samtools [34]. Visualization of bigwigs for genome tracks were generated from bam files using deeptools [35]. Quantification of properly aligned read pairs was done using the Rsubread package [41] in R (v.4.3.2) and the Ensembl GRCm38 release 102 annotation. Bioconductor package edgeR [42] (v. 3.42.4) was used to normalize counts across libraries and FPKMs were exported for plotting. Identification of differentially enriched genes (DEGs) between genotypes was performed using edgeR where DEGs were defined as having an FDR < 0.05 and an absolute log2FC > 0.5. Volcano plots were created in R using the Bioconductor package EnhancedVolcano (v. 1.18.0) and genotypes were overlayed using Adobe Photoshop. Heatmaps were generated from FPKMs of DEGs using Perseus [39] where rows were z-scored and hierarchical clustering was performed using average linkage and Euclidean distance. Pathway analysis of DEGs was done using Ingenuity Pathway Analysis (IPA; Qiagen) and heatmaps were made using GraphPad Prism (GraphPad Software, Inc., La Jolla, CA, v10). All Venn diagrams were made using Venny [38].

#### PRC2 overrepresentation analysis

PRC2 gene lists were obtained from ChIP Atlas [43]. Lists for target genes of Suz12, Eed and Ezh2 in mus musculus were used ± 1k from TSS. Lists were filtered for mean peak signal score ≥ 25 and compared to differential analysis result lists from RNA, ChIP and methylation. The expected fraction was calculated as the number of PRC2 genes (*n* = 8507) over the number of all genes (*n* = 55204) and the observed fraction was calculated as the number of PRC2 differentially enriched genes over the number of total differentially enriched genes for each individual comparison. The observed/expected ratio was then calculated as the observed fraction over the expected fraction. P values were determined using Fishers exact test.

#### Correlation analysis

Log2FPKM values from RNA-seq were calculated from the normalized count matrix with edgeR correcting for gene length. Transcription start sites (TSS) for all genes in the RNA count matrix were extracted from biomaRt in R and ChIP-seq reads were counted over 1000 bp bins centered on the TSS using bedtools (v2.27.1). CPMs were calculated using edgeR and log2 read values were used to compute Pearson’s correlation coefficient and generate scatterplots using the R CRAN package ggpubr/ggplot2 (v.0.6/v.3.5).

#### MAGMA gene set analysis

The gene set analysis tool MAGMA (v.1.10, de Leeuw, 2015) was utilized to compare genetic marker ensembles of human phenotypes to the RNA-seq results. First SNPs were annotated to genes using the GRCh37 (100 kb upstream, 20 kb downstream). Gene sets were created from genes showing a fold-change beyond ± 0.2 and a P. value ≤ 0.05 in KOs compared to WT. Mouse genes were annotated to human using a combination of biomaRt (v. 2.56.1) and NCBI tools (datasets version 16.26.0) where on average 88.3% of genes across all gene sets were mapped to human orthologs. Summary statistics for GWAS data was obtained for cognitive performance [44] based on 257,828 individuals, intelligence [45] based on 269,867 individuals, hippocampal volume [46] based on 21,297 individuals, bipolar disorder [47] based on 398,495 individuals, stroke [48] based on 524,354 individuals, dementia [49] based on 362,647 individuals, autism spectrum disorder [50] based on 18,382 individuals, and type 2 diabetes [51] based on 659,319 individuals. Gene-wise metrics were calculated for each gene in the GWAS data using the SNP-wise mean model, which aggregates SNP-level association statistics using the mean of all SNPs mapped to the same gene. These metrics are then adjusted for linkage disequilibrium using the European panel of the 1000 Genome data as a reference panel producing a gene-level p-value within the GWAS dataset. Competitive gene set analysis was then performed testing the association of genes within the KO gene sets to genes within the GWAS datasets to identify a stronger association with phenotype associated genes over non-associated genes.

### Statistical analysis

Statistical analysis was conducted as indicated in the figure legends using GraphPad Prism (GraphPad Software, Inc., La Jolla, CA, v10). Statistical significance was determined by two-tailed Student’s t-test and one-way ANOVA with Šídák´s multiple comparison test. Data represents mean ± SEM of at least three independent biological replicates. No statistical methods were used to predetermine sample size. The data distribution was assumed to be normal, but it was not formally tested.

## Results

### Loss of DNA glycosylases impairs spatial but not associative long-term memory

**Fig. 1:**
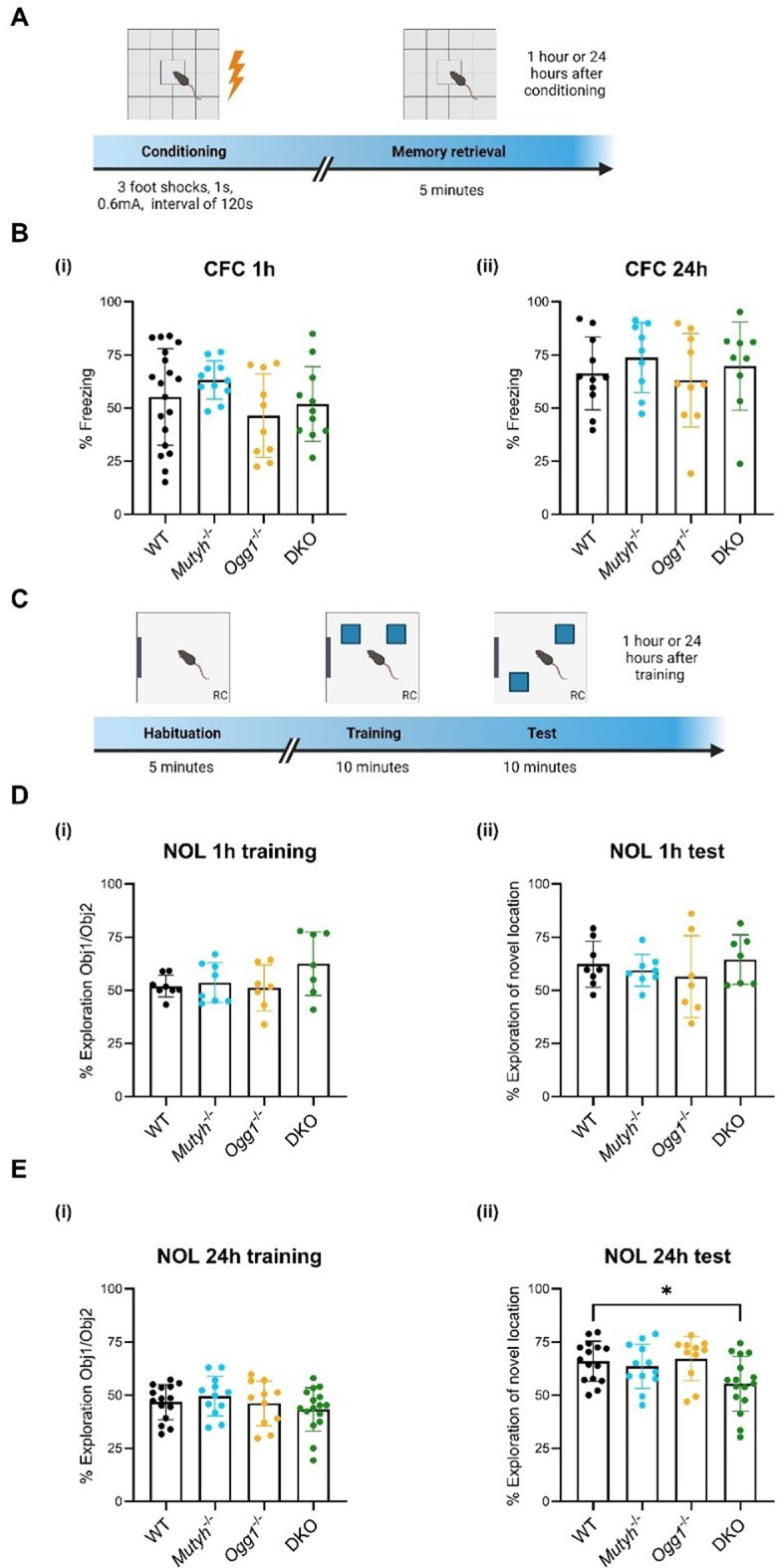
Loss of DNA glycosylases impairs spatial but not associative long-term memory (**A**) Experimental design of the contextual fear conditioning (CFC) paradigm. (**B**) Performance in the CFC task measured as freezing 1–24 h after training. At the 1 h (i) as well as at the 24 h (ii) training fear expression test *Mutyh*^-/-^, *Ogg1*^-/-^ and DKO mice showed similar levels of freezing when compared to wild-type (WT) (WT, *n* = 19; *Mutyh*^-/-^, *n* = 11; *Ogg1*^-/-^, *n* = 10; DKO, *n* = 11). (**C**) Experimental design of the novel object location (NOL) memory test. (**D**) (i) DNA glycosylase deficient and WT mice exhibited a similar preference for both objects during training. (ii) *Mutyh*^-/-^, *Ogg1*^-/-^ and DKO mice showed similar preference for the novel location and for the familiar location at the 1 h memory retention test compared to WT (WT, *n* = 8; *Mutyh*^-/-^, *n* = 8; *Ogg1*^-/-^, *n* = 7; DKO, *n* = 7). (**E**) (i) DNA glycosylase deficient and WT mice showed a similar preference for the two objects during the training phase. (ii) DKO mice exhibited impaired long-term location memory by spending less time exploring the novel object at the 24 h memory retention test compared to WT mice (WT, *n* = 15; *Mutyh*^-/-^, *n* = 12; *Ogg1*^-/-^, *n* = 11; DKO, *n* = 16). The relative exploration time is expressed as a percent discrimination index (D.I. = tnovel location / (tnovel location + tfamiliar location) × 100%). Data are presented as mean ± SEM, n values refer to the number of mice, *p < 0.05

To explore a potential involvement of DNA glycosylases Ogg1 and Mutyh in the regulation of short- and long-term memory, we employed DNA glycosylase-deficient mice, which have previously been characterized [13]. First, we subjected 6 months old male wild type (WT), single- (*Ogg1*^*-/-*^ or *Mutyh*^-/-^) and double-knockout (*Ogg1*^-/-^ x *Mutyh*^-/-^, DKO) mice to contextual fear conditioning (CFC), a hippocampus-dependent paradigm for associative learning (Fig. 1A). DNA glycosylase-deficient mice exhibited typical behavioral and physiological reactions during fear conditioning by displaying a normal response to electric foot shock exposure (Supplementary Fig. 1A) and normal fear acquisition (Supplementary Fig. 1B). Short-term and long-term contextual fear memory, assessed 1 h and 24 h after training respectively, remained unaffected in DNA glycosylase-deficient mice (Fig. 1B i and ii), suggesting that DNA glycosylases are not required for the consolidation of associative memories.

Next, we monitored WT, single-knockout and DKO mice in a novel object location (NOL) memory task (Fig. 1C). All mice displayed identical travel distances, indicating that motor function is not compromised in DNA glycosylase-deficient mice (Supplementary Fig. 2A). During the training phase, none of the genotypes showed a significant preference in percentage exploration time (Fig. 1D i and Fig. 1E i) and head entries (Supplementary Fig. 2B) for either object, suggesting that there is no evident bias towards one of the objects. In the memory retention test conducted 1 h after training, DNA glycosylase-deficient mice exhibited similar exploration of the novel object as WT, indicating that the loss of Ogg1 and/or Mutyh does not impair short-term memory (Fig. 1D ii). However, in the memory test conducted 24 h after training DKO mice exhibited a significant decrease in the exploration of the novel object location when compared to WT (Fig. 1E ii). Consistently, DKO mice exhibited no difference in head entries between the familiar and novel object location during the 24 h test (Supplementary Fig. 2C). This finding further supports the observation that DKO mice showed less interest in exploring the novel object location compared to other genotypes, indicating a memory deficit. Overall, our results suggest that both Ogg1 and Mutyh are required for long-term spatial memory that relies on spontaneous exploratory behavior.

### DNA glycosylases alter DNA methylation of polycomb repressive complex 2 (PRC2) target genes

Given that DNA glycosylases have previously been associated with DNA methylation [15, 16] and DNA methylation in the brain has been identified as a key regulator of memory consolidation [52, 53], we next asked whether Ogg1 and Mutyh affect memory formation by modulating DNA methylation.

**Fig. 2:**
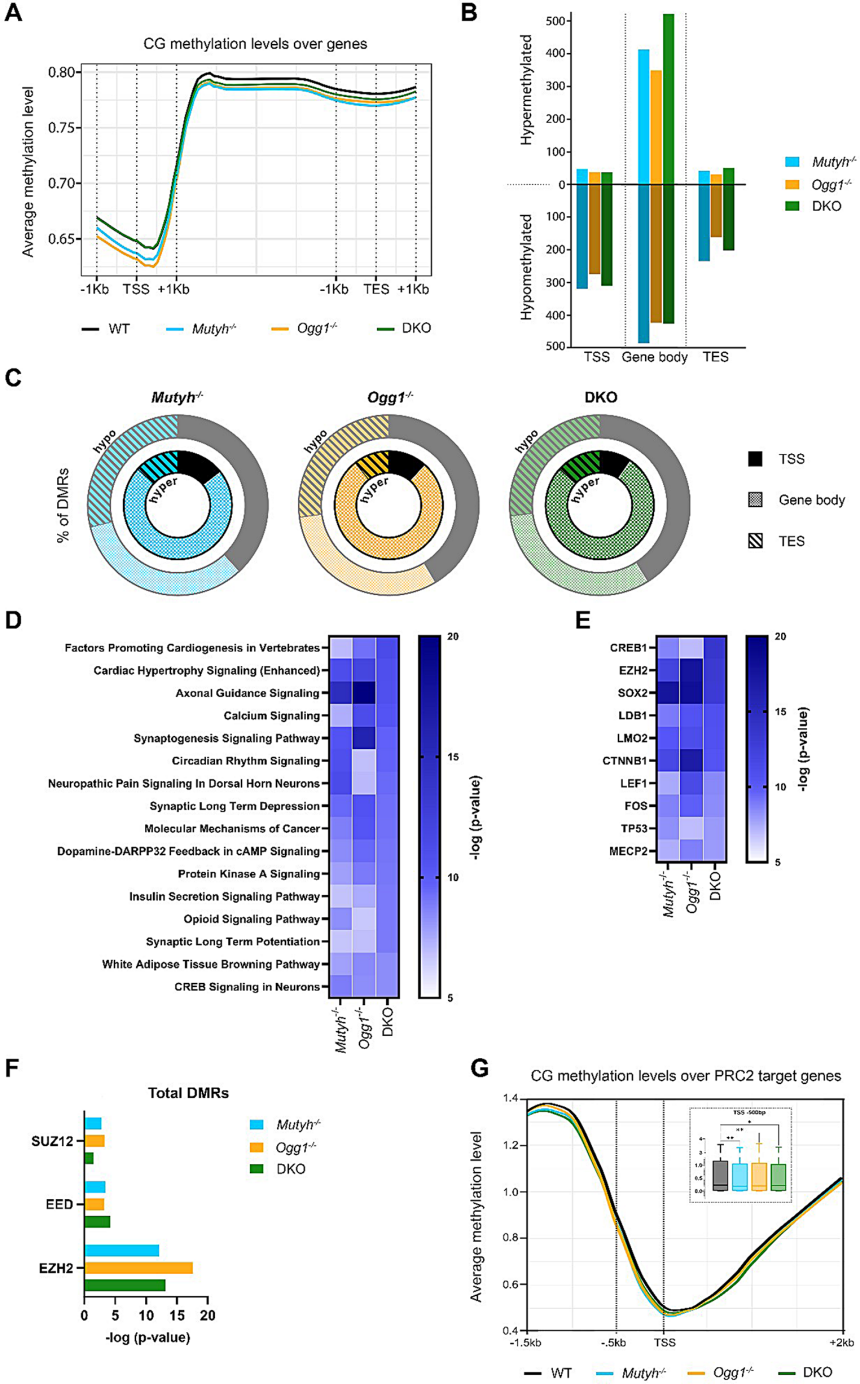
DNA glycosylases alter DNA methylation of PRC2 target genes. (**A**) Genome-wide average methylation level in the context of CG at transcription start site (TSS), gene body and transcription end site (TES). (**B**) Identification of differential methylation region (DMRs) and associated genes at TSS, gene body and TES. (**C**) Pie charts represent the percentage of hypomethylated (outer) and hypermethylated (inner) DMRs and associated genes at TSS, gene body and TES. (**D**) Pathways (-log10(p-value) < 0.05) identified by Ingenuity pathway analysis of total DMRs. Upstream regulators (-log10(p-value) < 0.05) identified by Ingenuity pathway analysis of (**E**) total DMRs and (**F**) total DMRs filtered for PRC2 subunits. (**G**) Average methylation level in the context of CG at the TSS of PRC2 target genes

We therefore performed whole-genome bisulfite sequencing (WGBS) on hippocampal DNA from 6 months old male WT and DNA glycosylase deficient mice to map single-cytosine methylation (5-methylcytosine, 5mC) levels genome-wide. Global mapping of 5mC to genomic locations revealed similar patterns between genotypes (Fig. 2A). However, CG methylation surrounding 5´ transcription start sites (TSS) were lower in *Ogg1*^-/-^ and *Mutyh*^-/-^ mice, whereas at gene bodies and transcription end sites (TES) CG methylation was reduced for both single knockout and DKO mice when compared to WT.

Next, we identified differentially methylated regions (DMRs) at specific genomic regions between DNA glycosylase deficient mice and WT hippocampus (Fig. 2B). We found 1542 regions with differential methylation in *Mutyh*^-/-^, 1279 regions in *Ogg1*^-/-^, and 1548 regions in DKO. Hypermethylated regions were predominantly located within gene bodies across all genotypes. However, the majority of DMRs across the genomic regions were hypomethylated. Interestingly, the highest percentage of hypomethylated regions was found at TSS, with up to 41% for both *Ogg1*^-/-^ and DKO (Fig. 2C). Subsequently, we examined whether DMRs identified at TSS, gene bodies, and TES would intersect among genotypes (Supplementary Fig. 3). The most notable overlap was detected for hypermethylated DMRs within gene bodies. The overlap for other DMRs across the genomic regions showed a moderate overlap between genotypes.

To understand the biological relevance and functions of altered DNA methylation we mapped all DMRs to their nearby gene annotation and performed ingenuity pathway analysis (IPA). As spatial memory impairment was found exclusively in DKO but not in single knockouts, pathways were filtered based on their significant enrichment in DKO. Among the top 16 most significant pathways for single and DKO we identified an enrichment for pathways that are related to key neuronal functions including axonal guidance signaling, synaptogenesis signaling pathway, synaptic long-term depression and potentiation and CREB signaling in neurons (Fig. 2D). In addition, targets of several upstream regulators that are key components of the nervous system, including CREB1, SOX2 and FOS, were significantly enriched in DMRs (Fig. 2E). Interestingly, enhancer of zeste homolog 2 (EZH2), an enzymatic component of the Polycomb Repressive Complex 2 (PRC2), was also found among the candidates with the most significant enrichment. In a previous study, we discovered a significant overlap between differentially expressed genes (DEGs) identified in the hippocampus of Ogg1- and/or Mutyh-deficient mice [13] and genes marked by H3K27me3, an epigenetic modification mediated by PRC2. Therefore, we examined whether DMRs across all genotypes were enriched for genes targeted by additional PRC2 subunits. In fact, we observed a significant enrichment of the PRC2 subunits suppressor of zeste 12 (SUZ12) and embryonic ectoderm development (EED), in addition to EZH2, among total DMRs across all genotypes (Fig. 2F). We then mapped the methylation signal to the TSSs of all identified PRC2 target genes and found a significant decrease in methylation across all knockouts in the 500 bp window immediately upstream of TSSs (Fig. 2G). Taken together, these results indicate that loss of Ogg1 and/or Mutyh alters DNA methylation at regulatory regions of PRC2 target genes in the adult hippocampus.

### DNA glycosylases mediate histone post-translational modifications and Suz12 occupancy

Based on these findings, we investigated next whether DNA glycosylases influence PRC2 function. PRC2 is a chromatin-associated protein complex that catalyzes the methylation of mono-, di-, and trimethylation of lysine 27 on histone H3 (H3K27). PRC2-mediated H3K27me3 leads to gene silencing and transcriptional repression important for maintaining cell fate, normal cellular functions and organismal development [54]. The core PRC2 subunits, SUZ12, EED, and the methyltransferase EZH2 or its closely related homolog EZH1, are all required for the catalytic activity of the complex [55, 56]. In addition, PRC2 is involved in the establishment and maintenance of bivalent chromatin domains that typically feature H3K27me3 alongside the methylation of H3 lysine 4 (H3K4me3), an active mark associated with gene transcription [57]. Hence, we examined the deposition of histone post-translational modifications (PTMs) that are associated with PRC2 function and the binding of the PRC2 subunit SUZ12.

**Fig. 3:**
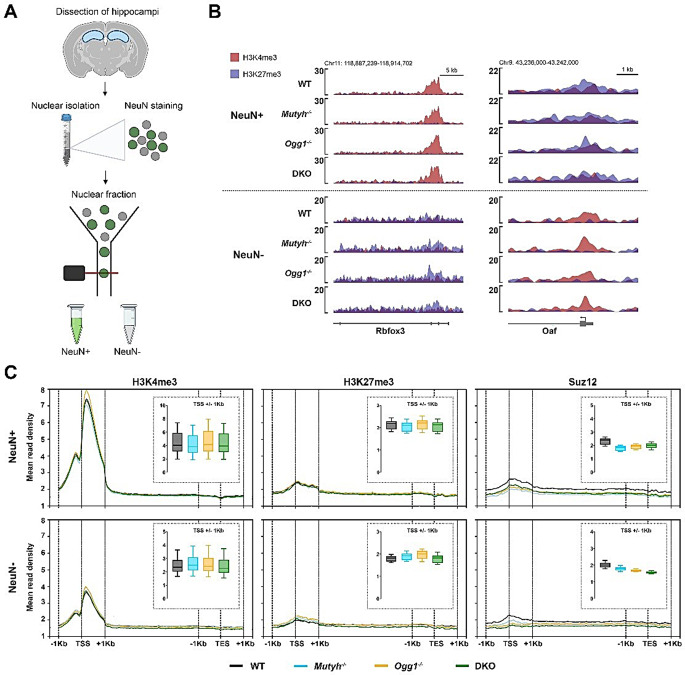
DNA glycosylases affect histone post-translational modifications and Suz12 occupancy in neurons and non-neuronal cells. (**A**) Experimental design for the isolation of cell-type specific nuclei used in next generation sequencing (NGS) approaches. (**B**) Confirmation of cell-type specific chromatin. Genome browser capture revealed an enrichment of representative H3K4me3 and H3K27me3 signals in NeuN + and NeuN- nuclei at a selected neuronal gene (Rbfox3) and a glial gene (Oaf). (**C**) Profile plots showing mean read distribution of H3K4me3, H3K27me3 and Suz12 over transcription start site (TSS) +/- 1000 bp, scaled gene body and transcription end site (TES) +/- 1000 bp. Boxplots represent the distribution of mean read density over the TSS +/- 1000 bp of all genes

To decipher DNA glycosylase-dependent cell-type specific epigenetic changes, we used fluorescence activated nuclear sorting (FANS) for hippocampal tissue and sorted for NeuN-positive (NeuN+) neuronal and NeuN-negative (NeuN-) non-neuronal nuclei [30] (Fig. 3A). Cell-type specificity was confirmed by the purity of the sorted cell populations and by immunocytochemistry (ICC) conducted after sorting (Supplementary Fig. 4A and 4B). Next, we generated ChIP-seq datasets for H3K4me3, H3K27me3, and Suz12 from NeuN + and NeuN- nuclei across all genotypes for three independent replicates. Chromatin signatures of cell-type specific genes demonstrated a successful enrichment for NeuN + and NeuN- nuclei (Fig. 3B). For instance, the neuronal gene Rbfox3 showed high levels of the activation mark H3K4me3 in NeuN + nuclei, while in NeuN- nuclei H3K4me3 signals were mostly absent and instead an enrichment for the repressive mark H3K27me3 was found. We observed no differences in the global genomic distribution of H3K4me3 or H3K27me3 among genotypes in either neurons or non-neuronal cells (Fig. 3C). However, the global genomic distribution of Suz12 occupancy at TSS within a range of plus or minus 1 kilobase (kb) was decreased in both NeuN + and NeuN- nuclei for all genotypes compared to WT.

We then examined the enrichment of H3K4me3, H3K27me3 and Suz12 across specific gene regions to gain deeper insights into their potential regulatory roles. We identified in total 15,897 consensus peaks for H3K4me3 (12627 in NeuN + and 3270 in NeuN-), 5827 consensus peaks for H3K27me3 (4131 in NeuN + and 1696 in NeuN-) and 1087 consensus peaks for Suz12 (775 in NeuN + and 312 in NeuN-). The genomic annotation of all consensus peaks combined showed that the majority of H3K4me3 peaks were located in promoters for both NeuN + and NeuN- nuclei (Supplementary Fig. 5A). For H3K27me3 and Suz12, consensus peaks were mostly identified at promoter and distal intergenic regions, similarly in neurons and non-neuronal cells. However, H3K27me3 demonstrated the highest number of consensus peaks in promoters of non-neuronal cells whereas the majority of Suz12 consensus peaks were located in promoters of neurons. The genomic location of consensus peaks across genotypes revealed a similar genomic distribution (Supplementary Fig. 5B). Moreover, the cell-type-specific distribution of consensus peaks revealed that the majority of peaks for H3K4me3, H3K27me3, and SUZ12 were present in NeuN + nuclei, with only a subset of peaks being specific for NeuN- nuclei, which was consistent across all genotypes (Supplementary Fig. 5C). Hierarchical clustering analysis of read densities showed a strong correlation among H3K4me3, H3K27me3, and Suz12 across cell-types, demonstrating a high reproducibility between our ChIP-seq replicates (Supplementary Fig. 6A). In summary, our data is of high technical quality and indicates that the loss of DNA glycosylases does not impact the global genomic distribution of H3K4me3 and H3K27me3 but alters the global allocation of Suz12 at TSS.

### DNA glycosylases regulate PRC2 associated histone PTMs at genes associated with cognitive function

**Fig. 4:**
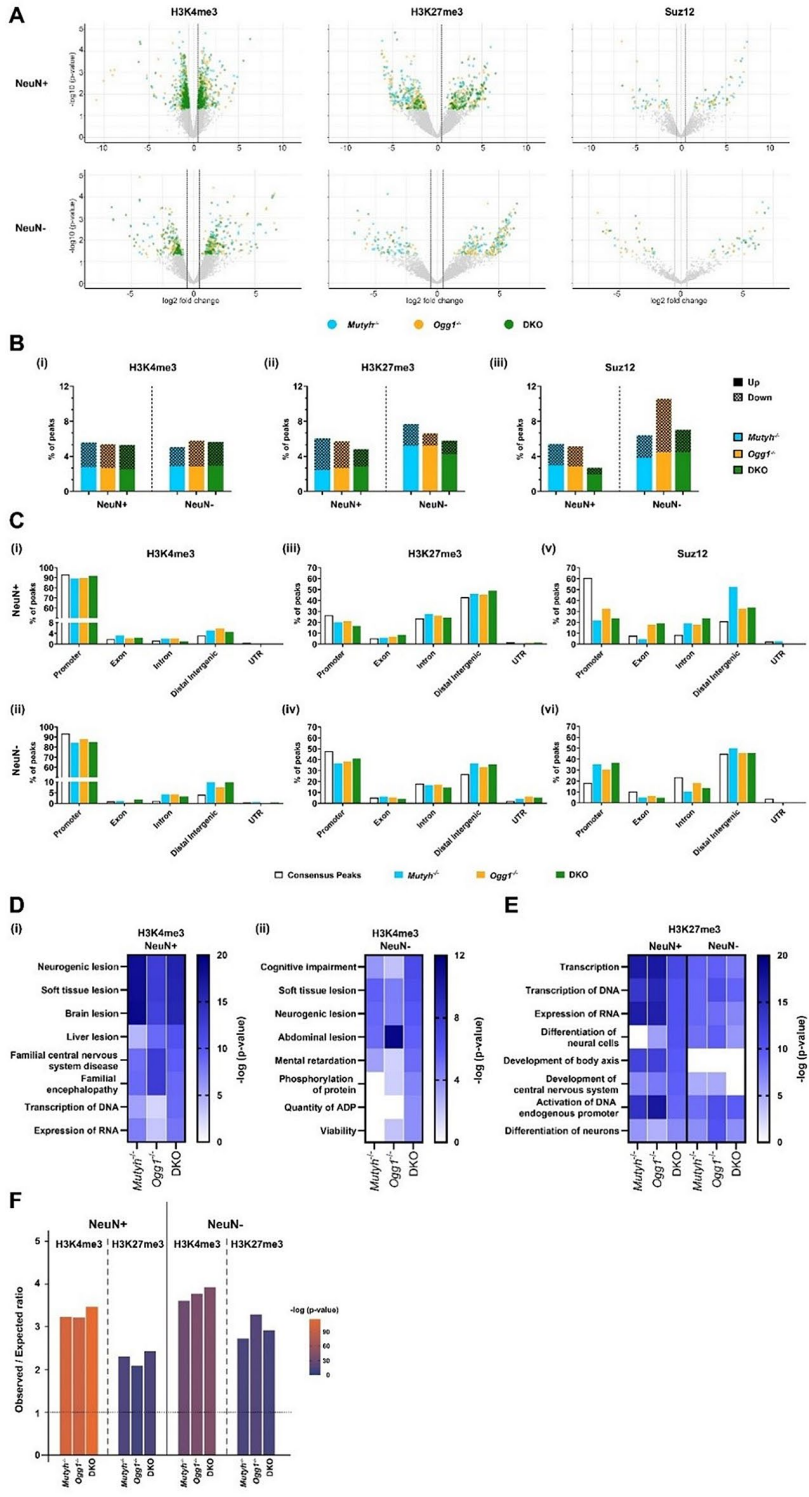
DNA Glycosylases mediate histone post-translational modifications associated with cognition in both neurons and non-neuronal cells. (**A**) Volcano plot representation of differential enriched regions (DERs) of H3K4me3 (i), H3K27me3 (ii) and Suz12 (iii) for NeuN + and NeuN- nuclei. (**B**) Percentage of DERs for H3K4me3 (i), H3K27me3 (ii) and Suz12 (iii) in either NeuN + or NeuN- nuclei across all genotypes. (**C**) Genomic annotations of DERs for H3K4me3 (i), H3K27me3 (ii) and Suz12 (iii) in either NeuN + or NeuN- nuclei of *Mutyh*^-/-^, *Ogg1*^-/-^ and DKO when compared to WT. (**D**) Disease and functions (-log10(p-value) < 0.05) identified by Ingenuity pathway analysis of DERs for H3K4me3 in neurons (i) and non-neuronal cells (ii). (**E**) Most significant disease and functions (-log10(p-value) < 0.05) identified by Ingenuity pathway analysis of DERs for H3K27me3 combined for neurons and non-neuronal cells. (**F**) Overrepresentation of PRC2 target genes in DERs of H3K4me3, H3K27me3 and Suz12 for NeuN + and NeuN- nuclei. Dashed lines indicate an observed / expected ratio ≥ 1

To uncover DNA glycosylase-specific alterations in histone PTMs and Suz12 occupancy, we identified differentially enriched regions (DERs) in our ChIP-seq datasets. In contrast to the global genomic distribution, we detected numerous region-specific alterations in histone PTMs and Suz12 occupancy (Fig. 4A). The total number of DERs for both over- and under-enriched regions were similar between single-knockouts and DKO mice for H3K4me3, H3K27me3 and Suz12, respectively (Supplementary Fig. 6B). On average, we identified 866 DERs for H3K4me3, 342 DERs for H3K27Me3, and 59 DERs for Suz12 across single-knockouts and DKO in both cell-types. The percentage of DERs revealed a similar distribution of over- and under enrichment in both NeuN + and NeuN- nuclei across all genotypes, indicating that both neurons and non-neuronal cells are affected by the loss of DNA glycosylases (Fig. 4B). Only for Suz12, we found a higher percentage of DERs for *Ogg1*^-/-^ and DKO in non-neuronal cells compared to neurons (Fig. 4B iii). Similar to changes in DNA methylation, we observed a moderate overlap of DERs between genotypes across cell-types (Supplementary Fig. 7). However, most of the DERs were still unique for each genotype, suggesting that histone

PTMs and Suz12 occupancy are mainly regulated differently by *Mutyh*^-/-^ and/or *Ogg1*^-/-^. Next, we analyzed the genomic distribution of DERs and found that in both NeuN + and NeuN- nuclei the majority of DERs for H3K4me3 were situated in promoter regions (Fig. 4C i and ii). In contrast, H3K27me3 DERs showed a more ubiquitous distribution and were located both at promoter regions and distal intergenic regions in NeuN + and NeuN- nuclei (Fig. 4C iii and iv). For Suz12 in neurons, we detected the highest number of DERs at distal intergenic regions across all genotypes (Fig. 4C v). In addition, we also found a high abundance of DERs at distal intergenic regions for Suz12 in NeuN- nuclei (Fig. 4C vi). Given that the majority of alterations for H3K27me3 also occurred at distal intergenic regions, these findings indicate a DNA glycosylase-dependent redistribution of Suz12, which consequently affects the genomic distribution of H3K27me3.

To comprehend the biological significance of differential H3K4me3, H3K27me3, and Suz12 occupancy, we mapped DERs to their nearby gene annotations and performed IPA (Fig. 4D and E). Since spatial memory impairment was only observed in DKO, pathways were ranked based on their significant enrichment in DKO. Among the most significant disease and function categories associated with H3K4me3 in neurons, pathways linked to various types of tissue damage, such as neurogenic lesions, soft tissue lesions, and brain lesions were identified across all genotypes (Fig. 4D I). Moreover, we found myelination signaling pathway and axonal guidance signaling as the top enriched canonical pathways in DKO (Supplementary Fig. 8A i). In non-neuronal cells, we found an enrichment of disease and functions associated with cognition including cognitive impairment and mental retardation (Fig. 4D ii). Unlike H3K4me3, the diseases and functions identified for H3K27me3 showed a strong overlap in both NeuN + and NeuN- nuclei. Interestingly, the majority of the top identified disease and functions were related to gene regulation including transcription, transcription of DNA, expression of RNA and activation of endogenous promoter in all genotypes (Fig. 4E). In non-neuronal cells, we identified synaptogenesis signaling pathway and activation of NMDA receptors and postsynaptic events as the highly enriched canonical pathways for H3K27me3, most significantly in DKO (Supplementary Fig. 8B ii). Because fewer DERs were identified for Suz12, representative pathway analysis could not be obtained.

To investigate whether PRC2 target genes are overrepresented in DERs of H3K4me3 and H3K27me3, we compiled a consensus list from publicly available ChIP-seq data in neuronal cells of Eed, Suz12 and Ezh2. The observed versus expected ratio showed a significant overrepresentation of PRC2 target genes in DERs of H3K4me3 and H3K27me3 for both NeuN + and NeuN- nuclei across all genotypes (Fig. 4F). Taken together, our data suggest that DNA glycosylase-dependent epigenetic changes are particularly prominent for PRC2 targets, which are associated with key functions and pathways relevant for cognition in both neurons and non-neuronal cells.

### DNA glycosylases mediate expression of PRC2 target genes important for neuronal function and signaling

**Fig. 5:**
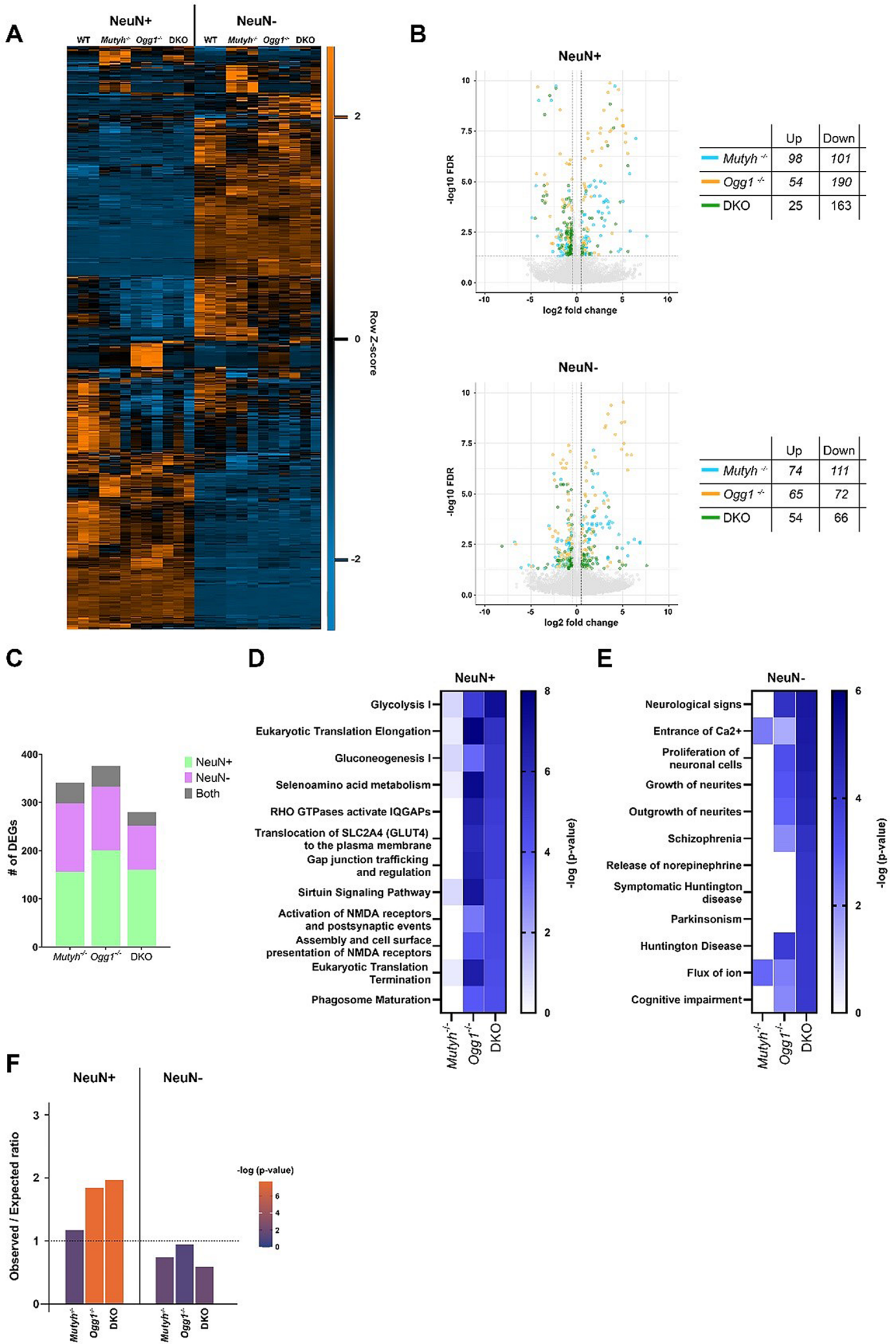
DNA glycosylases regulate gene expression important for neuronal function and signaling. (**A**) Heatmap over all differentially expressed genes (DEGs) (fold-change ≤ 0.5, FDR ≤ 0.05), clustered using Euclidean distance and average linkage. Values are FPKMs, z-scored by row across cell-type showing that DEGs are predominantly found in cell-type specific genes. (**B**) Volcano plot representation of DEGs across genotypes in NeuN + and NeuN- nuclei. Tables indicate the number of genes either up- or downregulated. (**C**) Cell-type specific DEGs across *Mutyh*^−/−^, *Ogg1*^−/−^ and DKO mice. DEGs were detected exclusively in either NeuN + or NeuN- cell populations, or in both. Pathways (-log10(p-value) < 0.05) identified by Ingenuity pathway analysis of DEGs in (**D**) neurons and (**E**) non-neuronal cells. (**F**) Overrepresentation of PRC2 target genes in DEGs for NeuN + and the NeuN-. Dashed lines indicate an observed / expected ratio ≥ 1

Next, we examined whether DNA glycosylase-dependent epigenetic changes affect gene expression in a cell-type specific manner by performing nuclear transcriptome profiling of NeuN + and NeuN- nuclei from DNA glycosylase-deficient and WT hippocampus. Cell-type specific RNA expression was confirmed through gene ontology analysis, which revealed neuronal processes primarily in NeuN + and glial-related processes in NeuN- nuclei (Supplementary Fig. 9A and 9B). Hierarchical clustering analysis of DEGs revealed that loss of Ogg1 and Mutyh predominantly affected genes with a cell-type specific expression profile (Fig. 5A). DEGs across all genotypes were primarily downregulated in both neurons (177 up and 454 down) and non-neuronal cells (193 up and 249 down), most prominently in *Ogg1*^-/-^ and DKO neurons (Fig. 5B). The majority of DEGs were either NeuN + or NeuN- specific while only a minority of genes was differentially expressed in both cell-types, indicating that DNA glycosylases regulate gene expression in a cell-type specific manner (Fig. 5C). Next, we investigated whether DEGs overlapped among genotypes and found again a moderate overlap of DEGs across all genotypes (Supplementary Fig. 10). The most pronounced overlap was observed in downregulated DEGs between *Ogg1*^-/-^ and DKO in neurons and non-neuronal cells.

IPA analysis of DEGs revealed a significant enrichment of several metabolic pathways, including glycolysis, gluconeogenesis, and selenoamino acid metabolism, which was particularly pronounced within neurons in *Ogg1*^-/-^ and DKO (Fig. 5D), suggesting Ogg1 as the driver of this effect. Interestingly, we also discovered pathways associated with NMDA receptors, with the strongest enrichment in DKO. Moreover, diseases and functions annotations showed a significant enrichment of pathways related to neurological disorders such as neuromuscular disease and Huntington disease (Supplementary Fig. 11A). In non-neuronal cells, we observed the most significant enrichment of DKO DEGs in diseases and functions related to the entrance of Ca2+, neurite growth, schizophrenia, Huntington’s disease, Parkinsonism, and cognitive impairment (Fig. 5E). Moreover, significant enriched canonical pathways included cAMP-mediated signaling, ion channel transport and interferon gamma signaling (Supplementary Fig. 11B). Interestingly, enrichment analysis using our compiled PRC2 gene list revealed a strong overrepresentation of DEGs from *Ogg1*^-/-^ and DKO in neurons, with no significant enrichment observed in non-neuronal cells (Fig. 5F).

In summary, while we observed cell-type specific alterations in gene expression following the loss of DNA glycosylases, DEGs in both cell-types were associated with highly relevant categories for neuronal function and cognition, particularly in DKO. Notably, PRC2 target genes were found to be primarily overrepresented among DEGs in neurons, indicating that DNA glycosylases regulate their expression in a cell-type specific manner.

### DNA glycosylases alter gene expression through modulating histone PTMs

**Fig. 6:**
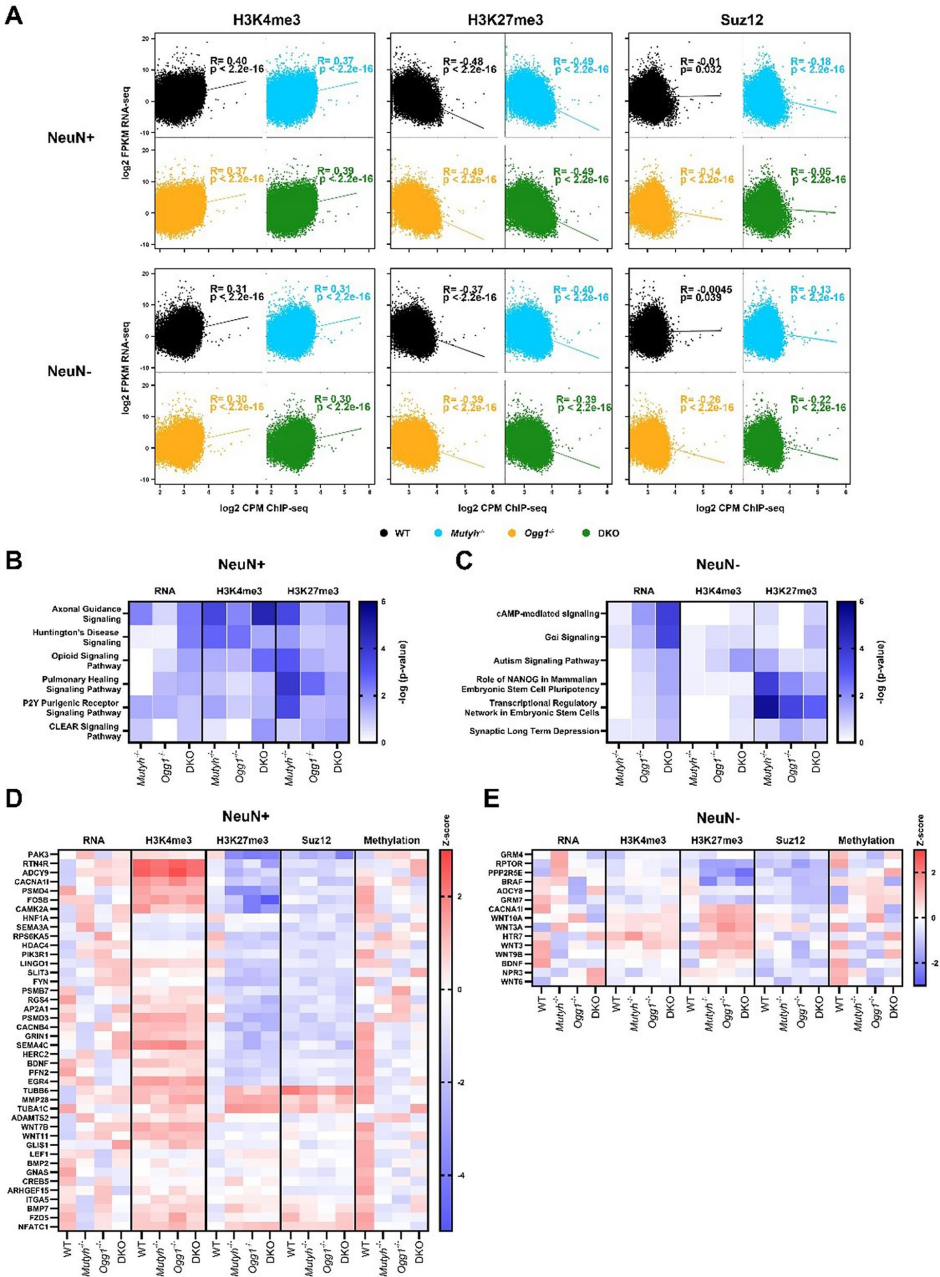
Histone post-translational modifications regulated by DNA glycosylases influence gene expression. (**A**) Spearman correlation analysis between ChIP-seq and RNA-seq data. Scatterplot showing the correlation of H3K4me3, H3K27me3, and Suz12 CPMs (counts per million) in the transcription start site (+/- 500 bp) with RNA FPKMs (fragment per kilobase million) for neurons and non-neuronal cells. (**B**) Overlapping pathways for RNA, H3K3me3 and H3K27me3 (-log10(p-value) < 0.05) identified by Ingenuity pathway analysis in neurons. (**C**) Overlapping pathways for RNA, H3K3me3 and H3K27me3 (-log10(p-value) < 0.05) identified by Ingenuity pathway analysis in non-neuronal cells. Differences in gene expression (FPKMs), DNA methylation (mean signal) and occupancy for histone post-translational modifications and Suz12 (CPMs in the TSS) for overlapping PRC2 target genes identified in the most significant pathways in (**D**) neurons and (**E**) non-neuronal cells

We then examined a possible connection between DNA glycosylase-dependent epigenetic modifications and transcriptional changes. Therefore, we performed Spearman correlation analysis between FPKMs (fragments per kilobase million) obtained through RNA-seq and TSS CPMs (counts per million) obtained from ChIP-seq (Fig. 6A). We observed a consistent positive correlation of H3K4me3 and RNA in neurons and non-neuronal cells across all genotypes. In contrast, H3K27me3, which is associated with transcriptional repression, demonstrated a consistent negative correlation with gene transcription. In addition, we observed a minor negative correlation between Suz12 occupancy and RNA expression for all genotypes and cell-types. To examine a potential correlation between DNA glycosylase-dependent DNA methylation and gene expression changes we intersected DMRs with DEGs and found only a minor overlap across all genotypes (Supplementary Fig. 12).

Since we only identified a minimal gene-specific overlap between DMRs, DERs and DEGs, we wondered whether similar biological processes are affected. Therefore, we intersected the pathways identified in each individual dataset that showed significant enrichment. Interestingly, the overlap revealed numerous pathways linked to critical neuronal processes and neurodegenerative diseases such as axonal guidance signaling, opioid signaling pathway and Huntington’s disease. Notably, DKO exhibited the strongest enrichment in axonal guidance signaling pathway for both RNA and H3K4me3 (Fig. 6B). In NeuN- nuclei, we found overlapping pathways related to key neuronal processes and diseases including cAMP-mediated signaling, synaptic long-term depression and autism signaling pathway, all showing the most significant enrichment in DKO for RNA (Fig. 6C). Next, we compared RNA expression (FPKMs), DNA methylation, Suz12 occupancy, and histone PTMs (CPMs) of genes identified in the overlapping neuronal process pathways across all genotypes, focusing on PRC2 target genes. We observed a negative correlation between RNA and H3K27me3 for genes targeted by PRC2 (such as GRIN1, HDAC4, and CAMK2A) in DKO neurons, while a positive correlation was found between RNA and H3K4me3 at PRC2 target genes (such as SEMAC4, GLIS1, and GRIN1) (Fig. 6D). Moreover, PRC2 target genes that showed higher RNA levels in DKO, including GRIN1, HDAC4, SEMA4C, and GLIS1, also displayed DNA hypomethylation in DKO neurons. In contrast, genes that appeared to be hypermethylated demonstrated a negative correlation with RNA levels in DKO neurons. In non-neuronal cells, we noticed a negative correlation between RNA levels and H3K27me3 for GRM7 and BDNF (Fig. 6E). Additionally, a decrease in GRM7 RNA levels correlated with DNA hypermethylation, whereas PRC2 target genes NPR3 and WNT6 with elevated RNA levels exhibited DNA hypomethylation.

Collectively, our findings suggest that DNA glycosylases Ogg1 and Mutyh regulate gene transcription by modulating the epigenetic landscape through histone PTMs and DNA methylation. Furthermore, our data indicate that DNA glycosylases regulate the expression of genes primarily targeted by PRC2, which are essential for neuronal function and signaling.

### DNA glycosylases affect expression of genes relevant for human cognitive function

To further explore a potential contribution of DNA glycosylase-regulated genes to human brain function, we examined their association with cognitive abilities and brain-related diseases in human genetic datasets. Consequently, we employed MAGMA, a fast and versatile tool for gene and gene-set analysis of genome-wide association studies (GWAS) data, to assess a potential enrichment of DNA glycosylase-regulated genes using GWAS summary statistics [58] from large-scale studies that aggregate data from multiple biobanks and disease-specific consortia.

**Fig. 7:**
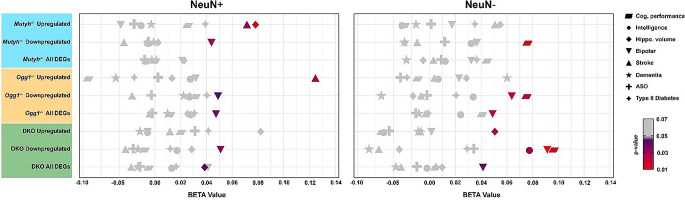
DNA glycosylase-regulated genes contribute to human cognitive abilities and bipolar disorder. MAGMA gene set analysis of cell-type specific differentially expressed genes in *Mutyh*^−/−^, *Ogg1*^−/−^ and DKO mice using summary statistics from multiple GWAS datasets. GWAS datasets are listed on the right: cognitive performance, intelligence, hippocampal volume, bipolar disorder, stroke, dementia, autism spectrum disorder (ASD) and type II diabetes. Symbols in color indicate a significant enrichment in the corresponding GWAS dataset (p-value < 0.05). DNA glycosylase-regulated gene sets are shown on the y-axis and BETA values (effect sizes) calculated by MAGMA are plotted on the x-axis. Full list of p- and BETA values are supplied in Supplementary Table 2

Intriguingly, we discovered that genes regulated by DNA glycosylases in both neurons and non-neuronal cells were significantly associated with cognitive traits and neurological diseases, but not with non-neurological diseases (Fig. 7). For instance, we found a significant enrichment of genes linked to hippocampal volume for all differentially expressed genes in DKO neurons and upregulated genes in DKO non-neuronal cells whereas for the single KOs only *Mutyh*^-/-^ upregulated genes showed a significant association. Cognitive performance was significantly associated with downregulated genes in *Mutyh*^*-/-*^, *Ogg1*^*-/-*^, and DKO non-neuronal cells, but with the highest enrichment in DKO. In addition, only in the downregulated genes in non-neuronal cells of DKO a significant enrichment of genes linked to human intelligence was found. Interestingly, we also detected a significant enrichment of genes associated with bipolar disorder across all genotypes but particularly among the downregulated genes in *Ogg1*^-/-^ and DKO neurons and non-neuronal cells, as well as in all differentially expressed genes in Ogg1 neurons and non-neuronal cells and DKO non-neuronal cells. Taken together, our findings uncover a previously undescribed role of DNA glycosylases beyond DNA repair by mediating gene expression relevant for human cognitive function and neuropsychiatric disorder.

## Discussion

While conventionally associated with preserving genome integrity, our findings suggest that DNA glycosylases Ogg1 and Mutyh play a significant role in altering the neuronal epigenome thereby contributing to the maintenance of cellular identities and spatial memory formation in the hippocampus. Our study demonstrates that Ogg1 and Mutyh play a role in regulating DNA methylation, particularly affecting the methylation status of PRC2 target genes. Moreover, we find that Ogg1 and Mutyh modulate histone modifications associated with PRC2 activity in neurons and non-neuronal cells of the hippocampus. Our results indicate that alterations in the epigenome, mediated by Ogg1 and Mutyh, correlate with the transcriptional regulation of predominantly PRC2 target genes, which are important for neuronal function and signaling underlying memory formation. Changes were found to primarily affect cell-type specific gene expression indicating that DNA glycosylases may have a role in maintaining cellular identities. Consistent with these findings, human genetic data revealed that epigenetic regulation of gene expression by DNA glycosylases contributes to human cognitive abilities and bipolar disorder.

### DNA glycosylases regulate spatial long-term memory

Both our research and other studies have highlighted the involvement of DNA glycosylases in higher brain functions associated with memory formation [59, 60]. In a previous study, we evaluated the learning and memory performance of single and double knockout mice deficient for Ogg1 and Mutyh by conducting a Morris water maze test. DKO mice were significantly slower in learning the position of the platform compared to wild-type mice but showed no difference in memory retention leaving the question if both Ogg1 and Mutyh affect memory formation unanswered [13]. Here, we demonstrate that the absence of Ogg1 and/or Mutyh does not influence short-term and long-term memory for contextual fear, suggesting that Ogg1 and Mutyh are not required for the consolidation of associative memories. In a novel object location memory task assessing spontaneous exploratory behavior, DKO mice exhibited no impairment in short-term memory but displayed a deficit in long-term spatial memory. Interestingly, we did not detect any memory formation impairment in Ogg1 and Mutyh single knockouts. However, the simultaneous deletion of both Ogg1 and Mutyh led to a deficit in memory retention, highlighting the importance of both DNA glycosylases in maintaining cognitive function. Our findings suggest that the presence of both Ogg1 and Mutyh is particularly important in memory tasks that recruit an innate behavioral preference for novelty and do not involve exogenous reinforcers such as swim stress or an electric foot shock. Consistent with our results, other studies demonstrate that stressful reinforcement stimuli might trigger alternative mechanisms that could compensate for the absence of both Ogg1 and Mutyh in our mouse model [61, 62].

### DNA glycosylases modulate DNA methylation and histone PTMs associated with PRC2 function

Oxidative DNA damage and its repair has been demonstrated to be involved in transcriptional regulation but whether these processes are directly linked has remained largely unexplored. We have previously shown that loss of Ogg1 and/or Mutyh impact gene transcription independent of global accumulation of 8-oxoG in the hippocampus [13]. However, the underlying mechanisms concerning how DNA glycosylases mediate gene expression independent of DNA repair remain elusive.

Here, we uncover a novel function for Ogg1 and Mutyh, beyond their traditional role in DNA repair, by modulating DNA methylation in the adult mouse hippocampus. DMRs across all genotypes were predominantly hypomethylated and at the TSS the majority of hypomethylated DMRs were significantly enriched in genes targeted by PRC2. Consistent with our findings, increasing evidence indicates that in other cell-types DNA glycosylases play an important role in modulating cellular function by influencing epigenetic alterations. Ogg1 and Mutyh have been demonstrated to bind to, but not to excise, 5mC, potentially preventing DNA demethylation [16, 63]. Interestingly, it has been shown that Ogg1 is part of a multiprotein complex that includes DNMT1 and EZH2, which facilitates CpG promoter methylation and induces alterations in histone PTMs such as H3K4me3 and H3K27me3^19^. Consistently, we observed that the absence of Ogg1 and Mutyh disrupts the occupancy of both H3K4me3 and H3K27me3 marks. The precise mechanism by which PRC2 is recruited to its target sites to promote transcriptional silencing remains unclear but our results suggest that Ogg1 and Mutyh may play a role in this process. In line with this, we demonstrated that PRC2 target genes were overrepresented among DERs of H3K4me3 and H3K27me3 in both neurons and non-neuronal cells, suggesting that DNA glycosylases regulate histone modifications predominantly associated with PRC2 activity. In addition, we found a redistribution of H3K27me3 and Suz12 upon loss of Ogg1 and Mutyh, indicating that PRC2 loses its ability to mediate the deposition of H3K27me3 at its target genes and occurs more randomly. We believe that the recruitment of PRC2 by Ogg1 and Mutyh is unlikely to occur through 8-oxoG. First, Mutyh recognizes adenine opposite 8-oxoG which is incorporated after replication [64]. Neurons, however, are post-mitotic and as such Mutyh is not required for 8-oxoG repair in those cells. Moreover, in a recent study, we profiled the genome-wide distribution of 8-oxoG and found no overlap of genes with 8-oxoG deposition and genes with altered expression in cells deficient for Ogg1 and Mutyh [25]. These findings suggest that 8-oxoG and DNA glycosylases modulate gene transcription independently and argue against an 8-oxoG-dependent mechanism. Alternatively, Ogg1 and Mutyh may recruit PRC2 to its target sites by recognizing secondary DNA structures such as G-quadruplex (G4) structures. G4 structures occur in CpG rich regions and have been implicated in gene regulation [65, 66]. Although the potential association between DNA glycosylases and G4 structures has been suggested [25, 67], further research is required to elucidate this potential mechanism.

### DNA glycosylases affect the expression of genes targeted by PRC2 and associated with human cognitive abilities

In the adult brain, PRC2 plays a crucial role in maintaining neuronal function and survival by transcriptionally silencing specific bivalent genes. Mice lacking PRC2 in neurons display signs of progressive neurodegeneration, with altered gene expression linked to Huntington’s disease [68]. In addition, it has been demonstrated that PRC2 is crucial for the activation of microglia and that loss of PRC2 causes changes in neuronal morphology and complex behaviors associated with neurodegenerative diseases [69]. In line with these findings, we found that upon loss of Ogg1 and Mutyh, cell-type specific alterations in gene expression were associated with highly relevant categories for neuronal function and cognition in both neurons and non-neuronal cells. Moreover, a previous study demonstrated that Ogg1 and Mutyh-dependent gene expression changes in a cancer cell line showed the highest enrichment in PRC2 target genes [25]. In the mouse hippocampus, a significant overlap between DEGs and genes harboring the epigenetic mark H3K27me3 has been reported [13]. Consistently, we provide evidence that PRC2 target genes were overrepresented among DEGs, suggesting that DNA glycosylases play a crucial role in regulating their expression. PRC2 is a well-known regulator of cellular differentiation and lineage specification throughout development where disruption of PRC2 regulation results in widespread phenotype changes and developmental delays [54]. Here, we find that the loss of DNA glycosylases results in only mild phenotype alterations and no obvious developmental delays indicating that their role lies in the long-term maintenance of epigenetic marks at PRC2 target genes and the preservation of cellular identity. Maintenance of the neuronal epigenome with age is important for cell-specific functionality and contributes to the integrity of cognitive processes like memory formation over time [70].

In addition, we discovered that gene expression changes within neurons were affecting various metabolic pathways. This association was evident in all DNA glycosylase-deficient mice, but most pronounced in Ogg1 and DKO suggesting that the effect is driven mainly by Ogg1. Our findings are consistent with previous studies indicating that Ogg1 plays a role in regulating cellular energy metabolism [71, 72]. Recent studies have demonstrated the importance of cellular metabolism in the intrinsic activity of neurons and memory formation [73, 74]. Consequently, the deficit in energy metabolism may partially contribute to the memory impairment seen in DKO mice. This is further supported by the strong association of gene expression changes with brain-related diseases and cognitive impairment upon loss of both Ogg1 and Mutyh. Importantly, several genes involved in these pathways are known PRC2 targets.

Notably, analysis of common GWAS variants associated with human cognitive traits and DNA glycosylase-regulated genes revealed an enrichment of genes linked to cognitive performance, intelligence and hippocampal volume. Hippocampal volume has been linked to various cognitive functions including processing speed, working memory, spatial navigation and abstract reasoning [75]. Interestingly, we identified the strongest enrichment of genes associated with cognitive performance among the downregulated genes in NeuN- nuclei of DKO, indicating that DNA glycosylases contribute to human cognition partly through glial cells. In the brain, all major types of glial cells have been found to impact neuronal signaling and synaptic transmission through various mechanisms [76–79]. A strong enrichment of common GWAS variants associated with bipolar disorder was identified across various DEG sets of single KOs and DKO. Bipolar disorders are a class of affect disorders characterized by biphasic episodes of mania and depression. Cognitive symptoms are highly prevalent in patients with bipolar disorder, emerging at the onset of symptoms and worsening as the disease progresses or becomes more severe [80]. Intriguingly, BER has been implicated in bipolar disorder, with studies showing that OGG1 is downregulated in a cohort of bipolar disorder patients [81]. Furthermore, a previous study demonstrated that single nucleotide polymorphisms (SNPs) in OGG1 and MUTYH are associated with depression, further emphasizing a significant role of DNA glycosylases in affect regulation and mental health [82]. Although the association with bipolar disorder was significant for DEG sets of single KOs and DKO, the most pronounced enrichment occurring in NeuN- nuclei of DKO, suggesting an additive effect resulting from the simultaneous depletion of Ogg1 and Mutyh.

In conclusion, our results indicate that Ogg1 and Mutyh cooperate with PRC2 to modulate the epigenome and, consequently, gene transcription in neurons and non-neuronal cells of the adult hippocampus. We therefore propose a novel role for Ogg1 and Mutyh beyond DNA repair in the epigenetic and transcriptional control of PRC2 target genes that are crucial for neuronal function and signaling. Notably, we observed the most pronounced molecular differences in DKO, which is in line with our finding that only the simultaneous deletion of Ogg1 and Mutyh results in impaired memory formation. In contrast, the absence of Ogg1 or Mutyh in single knockout mice showed no noticeable phenotypic effect on memory formation. Alterations in the epigenome and RNA expression occurred across all genotypes but varied in magnitude, suggesting that Ogg1 and Mutyh have an independent but synergistic effect on gene regulation underlying memory formation. Consistently, human genetic data revealed a previously undescribed link between DNA glycosylases and the etiology of higher cognitive functions and bipolar disorder. Hence, it will be intriguing to explore the role of Ogg1 and Mutyh in epigenetic maintenance underlying neurocognitive and psychiatric disorders. Future studies are needed to elucidate the full mechanisms by which DNA glycosylases Ogg1 and Mutyh cooperate with PRC2 to shape the epigenetic landscape in the brain. A better understanding of DNA glycosylases function in brain-related diseases could pave the way for novel therapeutic interventions.

## Electronic supplementary material

Below is the link to the electronic supplementary material.

Supplementary Material 1

## Data Availability

All genomic data produced in the present project (Bisulfite-seq data, ChIP-seq and RNA-seq) have been deposited in the NCBI GEO database under accession number GSE272603.
