# Supplementary material for: DNA glycosylases Ogg1 and Mutyh influence gene expression of PRC2 targets associated with cognition
Source: Cell Mol Life Sci. 2025 Aug 8;82(1):307. doi: 10.1007/s00018-025-05730-9 (PMC12334403; doi:10.1007/s00018-025-05730-9)
Supplement: Supplementary file 1 — Supplementary Material 1 [file 18_2025_5730_MOESM1_ESM.pdf]

Supplementary Material

DNA glycosylases Ogg1 and Mutyh influence gene expression of PRC2 targets associated with cognition

Andreas Abentung, Teri Sakshaug, Rabina Dumaru, Nina-Beate Liabakk, Mingyi Yang, Junbai Wang, Magnar Bjørås and Katja Scheffler

Supplementary table 1+2

Supplementary figures 1- 12

Antibody	Raised in	Company	#	Chromatin Input	Ab used per IP reaction
H3K27trime	mouse	Abcam	ab6002	2 µg	5 µg
H3K4trime	rabbit	Abcam	ab8580	2 µg	5 µg
Suz12	rabbit	Cell Signaling	#3737S	4.5 µg	1 µg

Supplementary Table 1. Antibody list and chromatin input for chromatin Immunoprecipitation

		Cognitive Performance		Intelligence		Hippocampal Volume		Bipolar		Stroke		Dementia		ASD		Type II Diabetes	
		Beta	Pval	Beta	Pval	Beta	Pval	Beta	Pval	Beta	Pval	Beta	Pval	Beta	Pval	Beta	Pval
NeuN+	DKO All DEGs	-0.008	0.607	0.013	0.331	0.039	0.041	0.041	0.052	-0.033	0.903	-0.024	0.812	-0.021	0.792	0.016	0.324
	DKO Downregulated	-0.014	0.662	0.017	0.297	0.029	0.115	0.051	0.033	-0.041	0.932	-0.024	0.790	-0.030	0.861	0.012	0.379
	DKO Upregulated	0.020	0.391	-0.010	0.555	0.082	0.065	-0.010	0.563	0.012	0.425	-0.021	0.629	0.031	0.315	0.041	0.330
	Mutyh-/- All DEGs	0.000	0.496	-0.013	0.699	0.023	0.102	0.021	0.150	-0.013	0.746	-0.003	0.546	-0.015	0.757	0.001	0.488
	Mutyh-/- Downregulated	-0.008	0.618	-0.010	0.636	0.002	0.451	0.044	0.030	-0.043	0.968	-0.001	0.519	-0.009	0.649	-0.011	0.630
	Mutyh-/- Upregulated	0.026	0.297	-0.019	0.659	0.078	0.012	-0.047	0.883	0.072	0.032	-0.006	0.554	-0.029	0.760	0.039	0.254
	Ogg1-/- All DEGs	0.012	0.359	0.028	0.186	0.025	0.141	0.048	0.038	-0.008	0.613	0.010	0.369	-0.004	0.560	0.031	0.216
	Ogg1-/- Downregulated	0.032	0.187	0.027	0.210	0.027	0.145	0.049	0.045	-0.032	0.869	0.022	0.240	-0.005	0.570	0.040	0.168
	Ogg1-/- Upregulated	-0.092	0.873	0.028	0.361	0.013	0.408	0.032	0.312	0.125	0.029	-0.055	0.781	0.002	0.486	-0.029	0.612
NeuN-	DKO All DEGs	0.005	0.437	-0.002	0.530	0.010	0.313	0.041	0.041	-0.040	0.955	-0.047	0.972	-0.018	0.771	0.005	0.443
	DKO Downregulated	0.096	0.014	0.077	0.031	-0.042	0.926	0.091	0.005	-0.070	0.979	-0.084	0.988	0.034	0.169	0.029	0.281
	DKO Upregulated	-0.066	0.958	-0.064	0.959	0.051	0.026	0.001	0.481	-0.013	0.666	-0.016	0.693	-0.056	0.964	-0.012	0.617
	Mutyh-/- All DEGs	0.045	0.058	0.012	0.327	-0.009	0.678	0.032	0.081	0.003	0.448	-0.033	0.908	0.009	0.354	0.037	0.129
	Mutyh-/- Downregulated	0.076	0.011	0.032	0.153	-0.035	0.937	0.037	0.086	-0.017	0.740	-0.035	0.896	0.012	0.337	0.035	0.176
	Mutyh-/- Upregulated	-0.034	0.752	-0.038	0.785	0.055	0.060	0.017	0.340	0.051	0.108	-0.019	0.666	0.001	0.490	0.035	0.274
	Ogg1-/- All DEGs	0.041	0.089	0.024	0.197	-0.003	0.556	0.049	0.022	-0.006	0.593	-0.015	0.727	-0.019	0.784	-0.002	0.529
	Ogg1-/- Downregulated	0.075	0.024	0.034	0.170	-0.023	0.812	0.064	0.020	-0.012	0.649	-0.067	0.981	-0.004	0.550	0.020	0.322
	Ogg1-/- Upregulated	-0.017	0.636	0.007	0.441	0.029	0.189	0.023	0.272	0.004	0.461	0.060	0.061	-0.041	0.857	-0.031	0.735

Supplementary Table 2. MAGMA gene set analysis including p- and BETA values

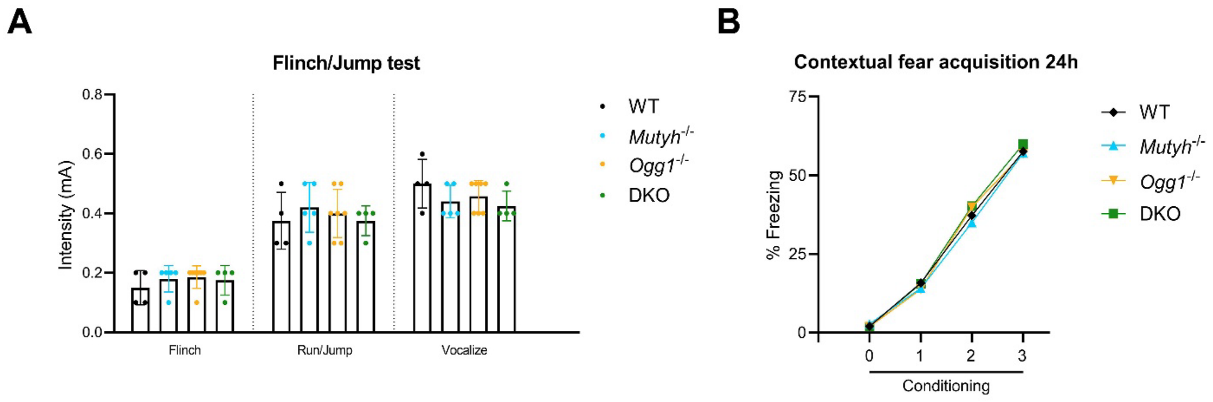

Supplementary figure 1. DNA glycosylase deficient mice show no abnormalities in the contextual fear paradigm

(A) DNA glycosylase deficient mice show a normal response to electric foot shock. Flinch/Jump test revealed similar general responses to electric foot shock between *Mutyh*$^{-/-}$, *Ogg1*$^{-/-}$ and DKO mice when compared to WT mice (WT, n = 4; *Mutyh*$^{-/-}$, n = 5; *Ogg1*$^{-/-}$, n = 7; DKO, n = 4). Mice were scored based on their first visible response to the foot shock (flinch), their first pronounced motor response (run or jump), and their first vocalized distress as described in Materials and Methods. Data are presented as mean ± SEM, n values refer to the number of mice. (B) In the contextual fear conditioning paradigm, DNA glycosylase deficient mice showed similar levels of freezing to WT during the fear-acquisition phase. Average freezing presented per genotype throughout conditioning (0=acclimatisation, 1,2,3=sequential electric foot shocks; WT, n = 11; *Mutyh*$^{-/-}$, n = 9; *Ogg1*$^{-/-}$, n = 10; DKO, n = 9; n values refer to the number of mice).

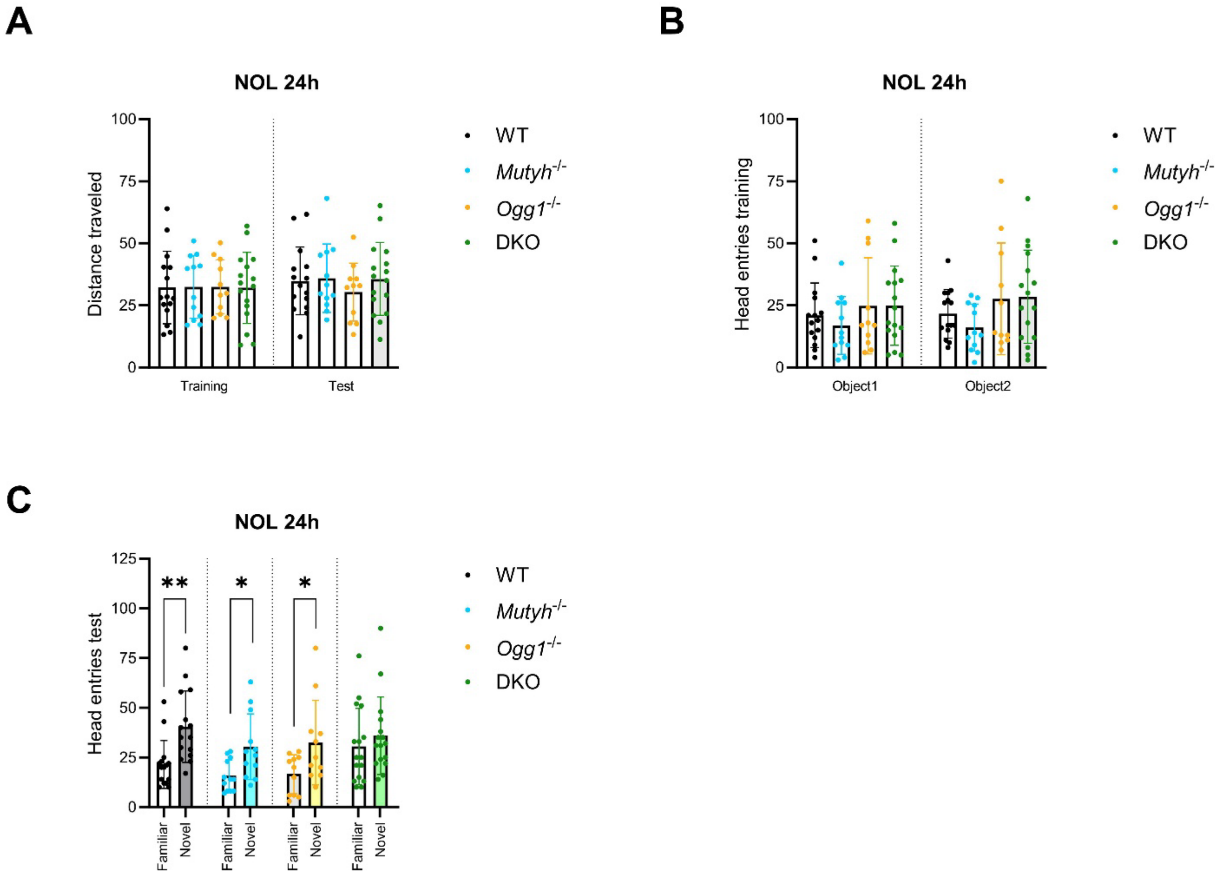

Supplementary figure 2. Novel object location (NOL) paradigm revealed a difference in head entries for DKO mice during the 24 hour NOL test

(A) Analysis of the distance traveled (in meters) during the novel object location paradigm revealed no difference between WT, *Mutyh*$^{-/-}$, *Ogg1*$^{-/-}$ and DKO mice during training and the 24 hours test (WT, n = 15; *Mutyh*$^{-/-}$, n = 12; *Ogg1*$^{-/-}$, n = 11; DKO, n = 16; n values refer to the number of mice). (B) Head entries examined during training indicated that DNA glycosylase deficient and WT mice (n = 15) had no preference for either of the objects (*Mutyh*$^{-/-}$, n = 12; *Ogg1*$^{-/-}$, n = 11; DKO, n = 16). (C) During the NOL test for 24 hours WT, *Mutyh*$^{-/-}$, *Ogg1*$^{-/-}$ showed a significant increase in head entries for the novel object. Head entries examined for DKO mice revealed no difference between the familiar and the novel object (Student's t-test, WT, n = 15, p = 0.002; *Mutyh*$^{-/-}$, n = 12, p = 0.0114; *Ogg1*$^{-/-}$, n = 11, p = 0.0355; DKO, n = 16, p = 0.4379). Data are presented as mean ± SEM, n values refer to the number of mice, *p < 0.05.

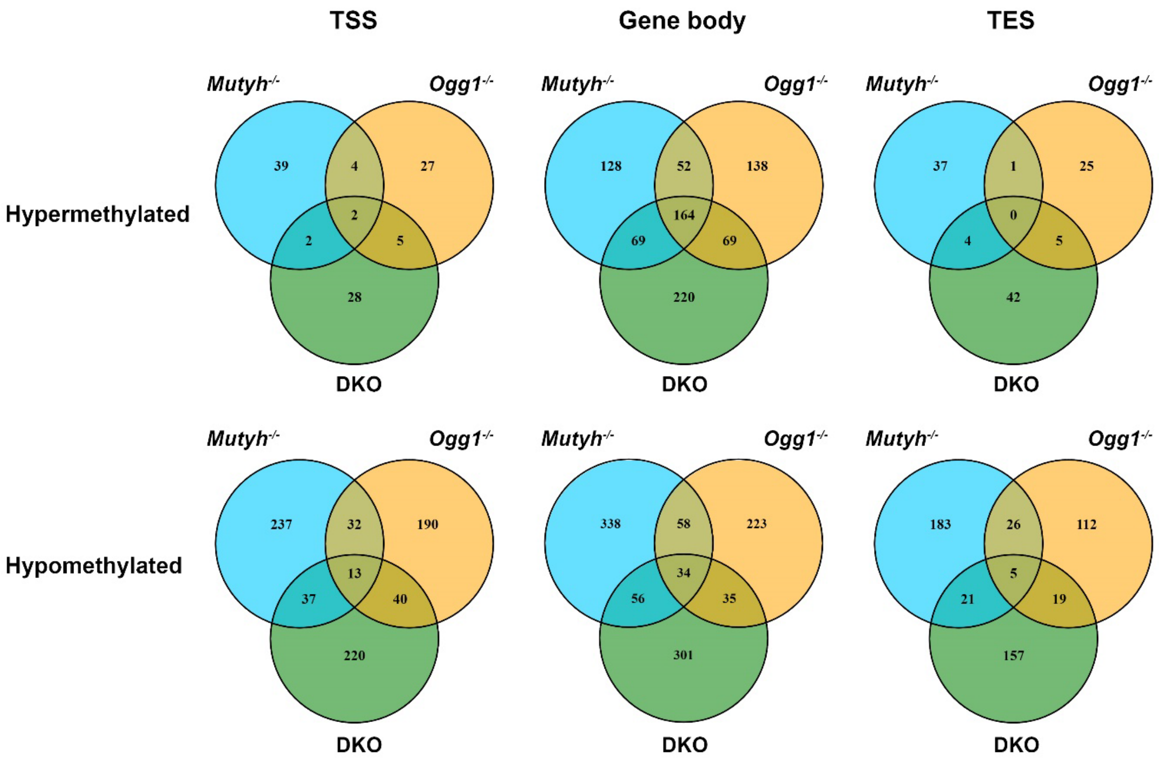

Supplementary figure 3. Overlap of DNA Methylation across genotypes

Venn diagrams depict the overlap of DNA methylation among genotypes for either hyper- or hypomethylated sites at the transcription start site (TSS), gene body and transcription end site (TES).

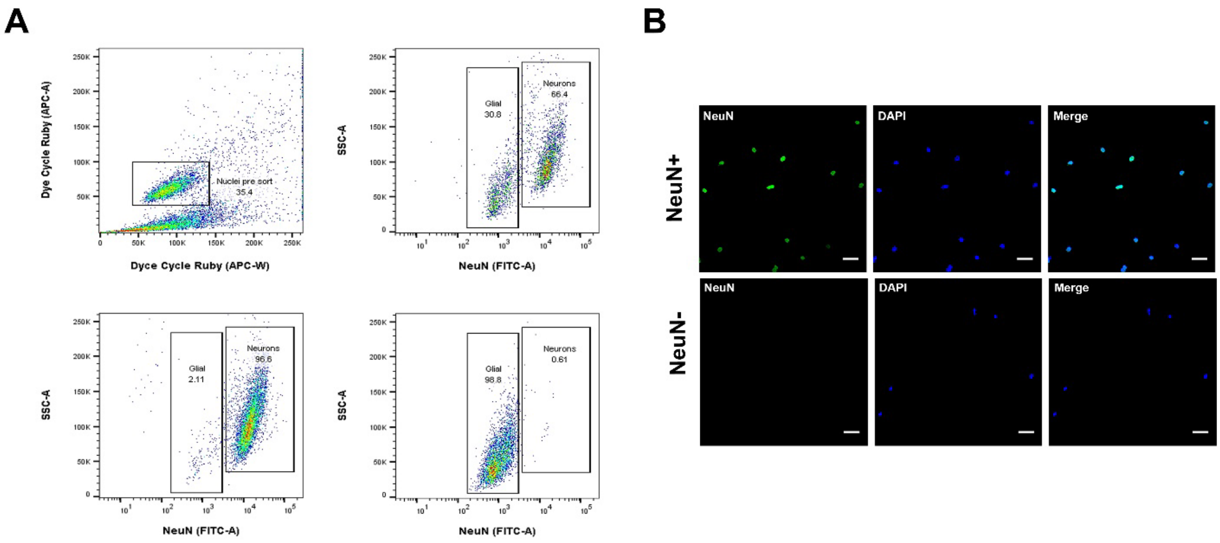

Supplementary figure 4. Fluorescence activated nuclear sorting (FANS) of cell type-specific nuclei

(A) FANS profiles for Dye cycle Ruby and NeuN demonstrating the gating strategy during the sorting (top). Purity of the sorting process was confirmed by probing the sorted cell populations after sorting (bottom). Percentage of the filtered population are indicated within the channel. Similar results were obtained for all three independent experiments. (B) Confocal images of nuclei to confirm the purity of NeuN+ and NeuN- nuclei after sorting (Scale bar = 30 µM).

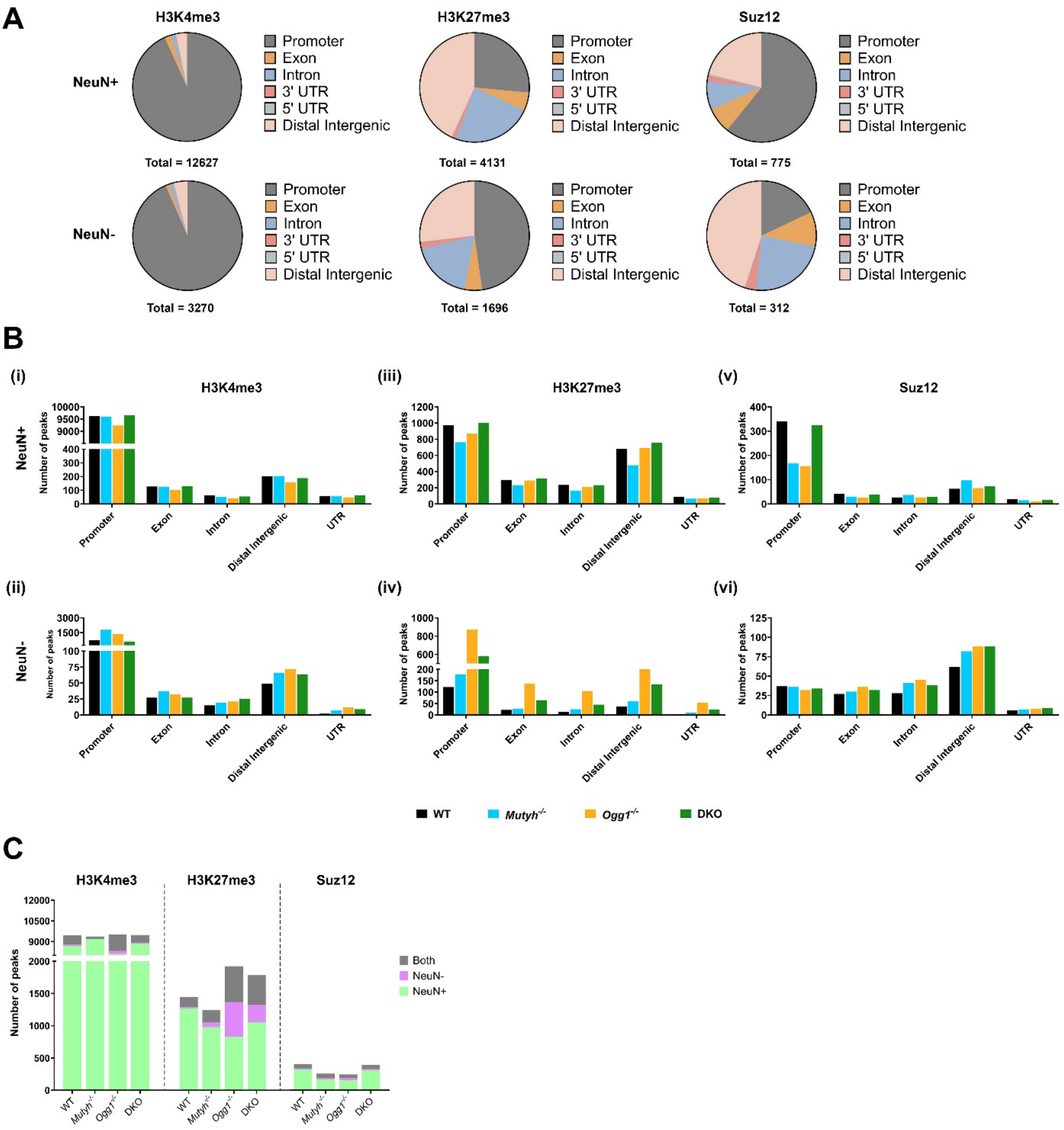

Supplementary figure 5. Characterization of identified consensus peaks

(A) Pie charts demonstrating the genomic annotation of total consensus peaks. (B) Genomic annotations of consensus peaks for H3K4me3 (i), H3K27me3 (ii) and Suz12 (iii) in either the NeuN+ or NeuN- populations of WT, *Mutyh*$^{-/-}$, *Ogg1*$^{-/-}$ and DKO. (C) Cell-type specific distribution of consensus peaks for H3K4me3, H3K27me3 and Suz12 across the hippocampus of WT, *Mutyh*$^{-/-}$, *Ogg1*$^{-/-}$ and DKO mice.

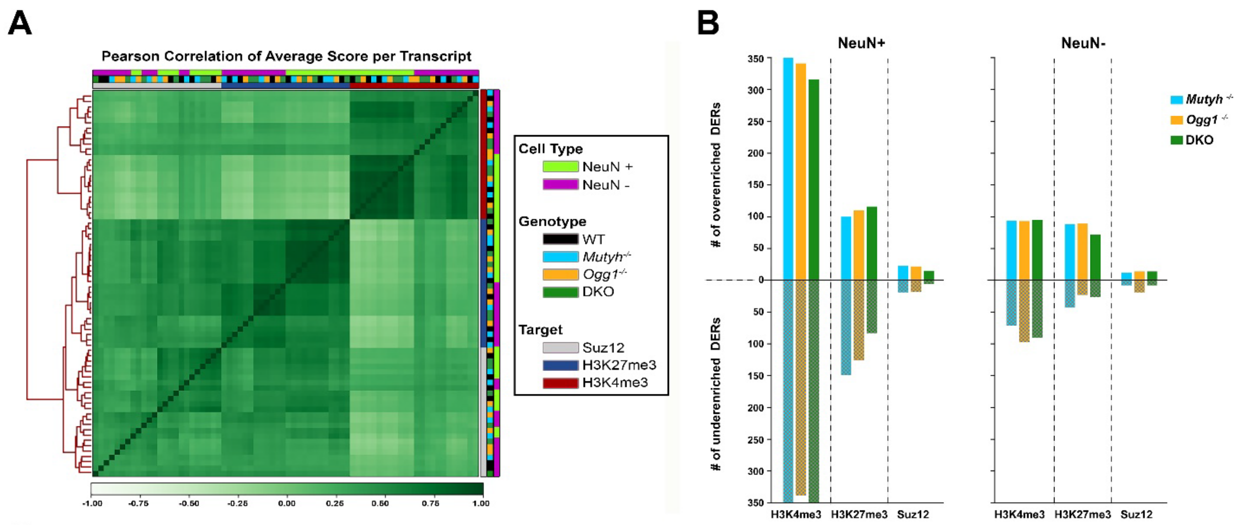

Supplementary figure 6. Characterization of H3K4me3, H3K27me3 and Suz12 occupancy

(A) Correlation heatmap with hierarchical clustering of read densities for H3K4me3, H3K27me3 and Suz12 replicates for NeuN+ and NeuN-. (B) Number of differentially enriched regions for H3K4me3, H3K27me3 and Suz12 either over-enriched or under-enriched for NeuN+ and NeuN-.

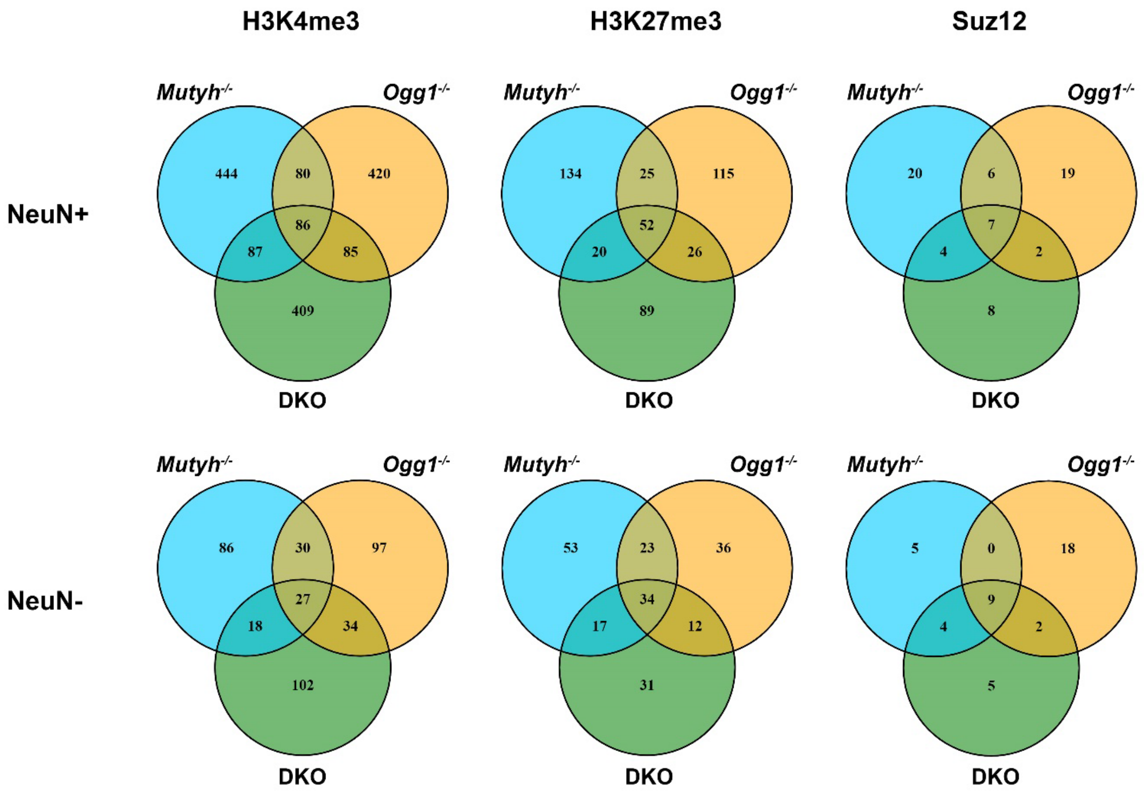

Supplementary figure 7. Overlap of H3K4me3, H3K27me3 and Suz12 occupancy across genotypes

Venn diagrams illustrate the intersection of H3K4me3, H3K27me3, and Suz12 occupancy across genotypes for both neurons and non-neuronal cells.

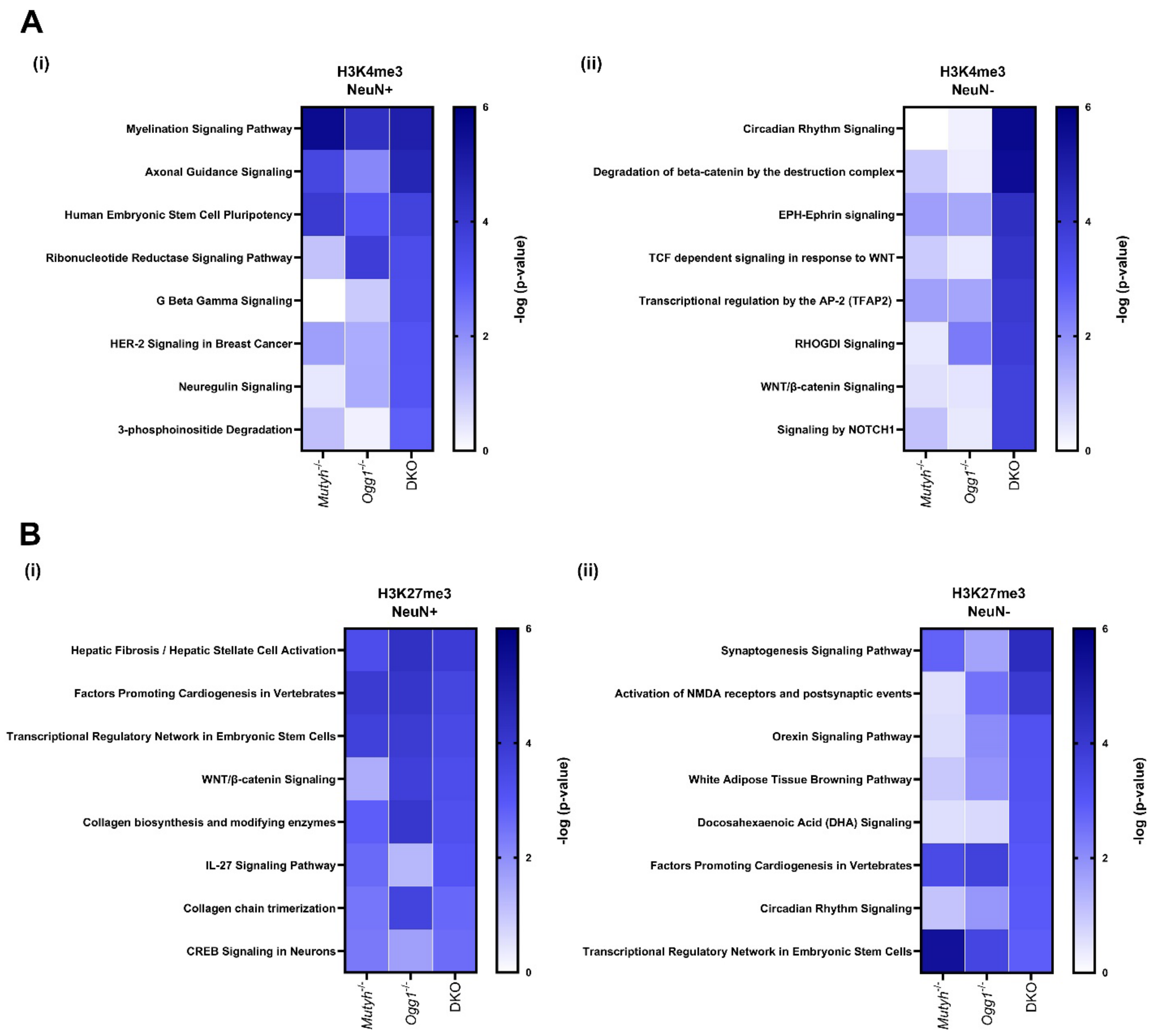

Supplementary figure 8. Pathways for differentially enriched regions for H3K4me3 and H3K27me3

(A) Pathways (-log10(p-value) <0.05) identified by Ingenuity pathway analysis of DERs in neurons. (B) Pathways (-log10(p-value) <0.05) identified by Ingenuity pathway analysis of DERs in non-neuronal cells.

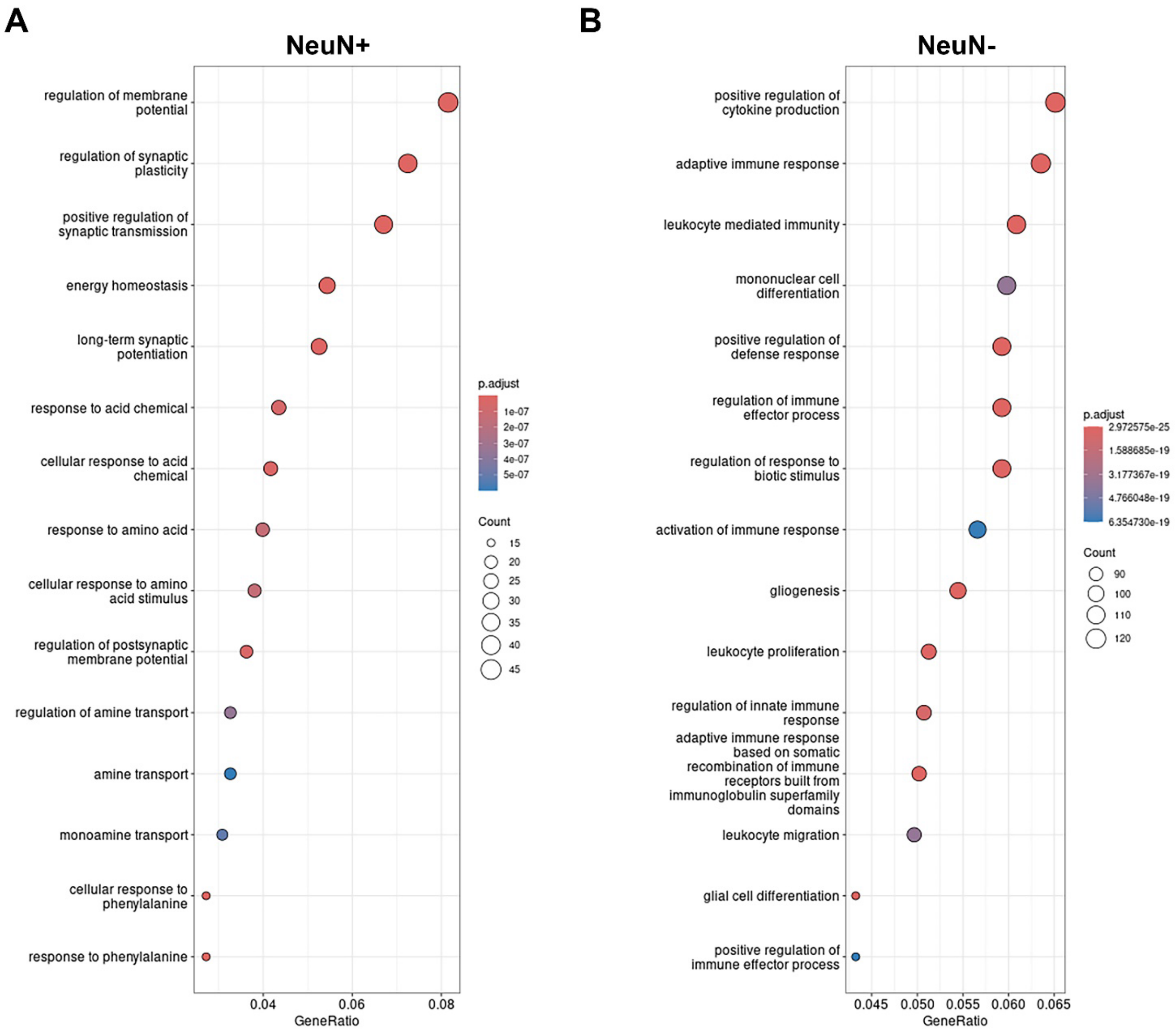

Supplementary figure 9. Isolation of cell-type specific nuclei by fluorescence activated nuclear sorting (FANS) for RNA-sequencing

Gene ontology analysis revealed cell-type specific RNA expression in neurons (A) and non-neuronal cells (B) in wild type mice.

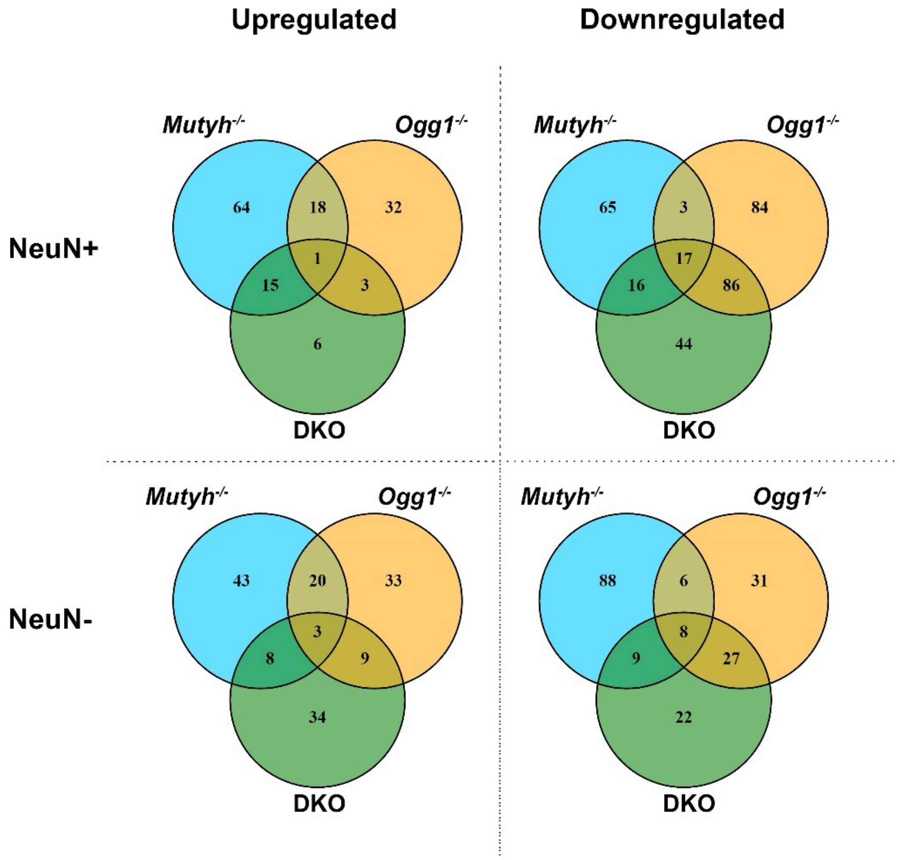

Supplementary figure 10. Overlap of differentially expressed genes across genotypes

Venn diagrams depict the overlap of differentially expressed genes that are either up- or downregulated among genotypes in NeuN+ or NeuN-.

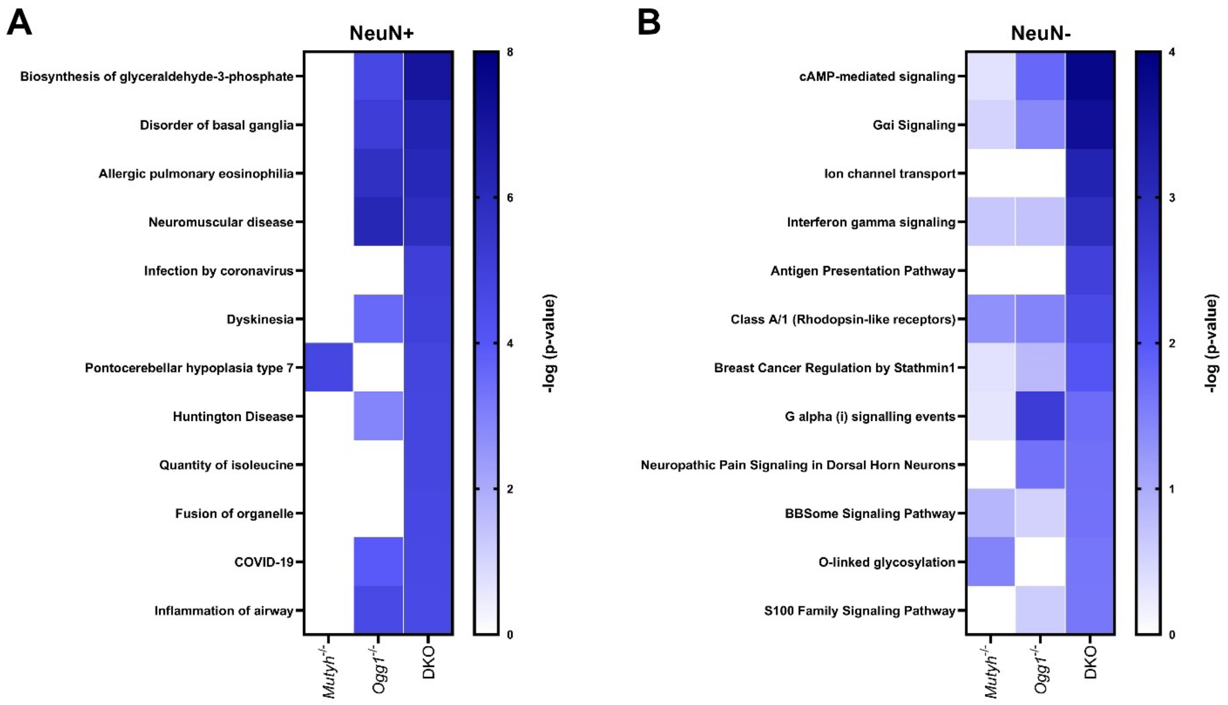

Supplementary figure 11. Pathways analysis for differentially expressed genes

(A) Pathways (-log10(p-value) <0.05) identified by Ingenuity pathway analysis of differentially expressed genes (DEGs) in non-neuronal cells. (B) Disease and functions (-log10(p-value) <0.05) identified by Ingenuity pathway analysis of DEGs in neurons.

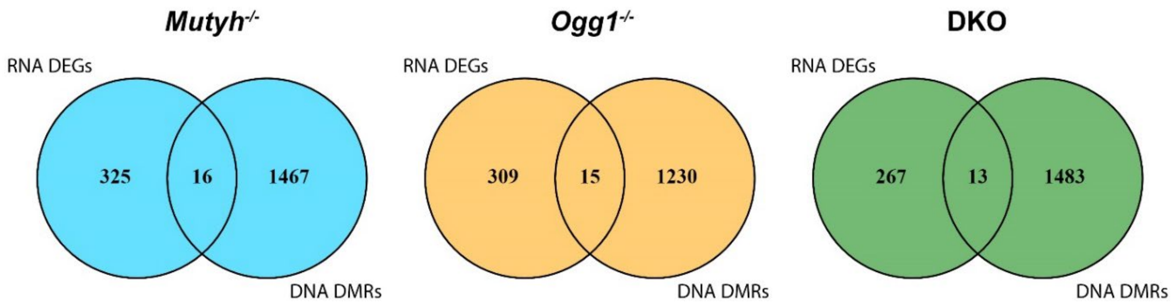

Supplementary figure 12. Correlation between RNA expression and DNA methylation across genotypes

Venn diagrams illustrate the overlap of combined gene expression changes (NeuN+ or NeuN-) and DNA methylation for each genotype.
